# Harnessing faba bean MAGIC populations for enhanced protein content, yield, and agronomic performance in diverse environments

**DOI:** 10.3389/fpls.2026.1731294

**Published:** 2026-02-23

**Authors:** Lynn Abou Khater, Fouad Maalouf, Cassandra Walker, Outmane Bouhlal, Khawla Aloui, Rind Balech, Kaoutar El Mahmoudi, Mohammed Ibriz, Francis Chuks Ogbonnaya, Shiv Kumar

**Affiliations:** 1 Biodiversity and Crop Improvement Program (BCIP), International Center for Agricultural Research in the Dry Areas (ICARDA), Terbol, Lebanon; 2 Agriculture Victoria, AgriBio, Centre for AgriBioscience, Horsham, VIC, Australia; 3 Biodiversity and Crop Improvement Program (BCIP), International Center for Agricultural Research in the Dry Areas (ICARDA), Rabat, Morocco; 4 Faculté des Sciences, Ibn Tofail University, Kenitra, Morocco; 5 Grains Research and Development Corporation, Canberra, ACT, Australia; 6 Biodiversity and Crop Improvement Program (BCIP), International Center for Agricultural Research in the Dry Areas (ICARDA), New Delhi, India

**Keywords:** faba bean, grain yield, MAGIC population, protein content, trait analysis

## Abstract

Faba bean (*Vicia faba* L.) is an increasingly important source of plant-based protein; however, improving both grain yield and protein content remains a major breeding challenge. This study aimed to identify superior haplotypes combining these traits using a large multi-parent advanced generation inter-cross (MAGIC) population. A total of 2,431 MAGIC lines were developed through two rounds of eight-way F1 inter-crossing among 28 parental lines and evaluated during the 2023/2024 growing season at Terbol, Lebanon, and Marchouch, Morocco, using an augmented design with four repeated checks. Phenological and yield-related traits were recorded, and protein content was quantified using both the Kjeldahl method and Near-Infrared Spectroscopy (NIRS; FOSS DS2500). The grain-based calibration model developed using modified partial least squares regression demonstrated high predictive accuracy (R² = 0.983), with cross-validation and training/test split confirming its reliability (R² = 0.82). Flowering and maturity periods were longer at Terbol, where significantly higher numbers of pods and seeds per plant, grain yield, and biological yield were also observed. Protein content ranged from 12.0% to 30.4% and showed a strong positive correlation between locations (r = 0.6, *p* < 0.001), indicating high repeatability across environments. Notably, protein content was not correlated with grain yield, highlighting the potential for simultaneous improvement of both traits. Ten transgressive lines exhibiting at least 25% higher protein content and grain yield than the parental checks were identified across sites. These findings demonstrate the effectiveness of MAGIC populations for capturing genetic variability and accelerating the development of high-yielding, protein-rich faba bean cultivars, providing valuable resources for future breeding programs.

## 1 Introduction

The rapidly growing global population, combined with climate change, is placing unprecedented pressure on food production systems, raising major concerns related to food security, greenhouse gas emissions, and environmental sustainability. In response, diversification toward alternative plant-based protein sources has become increasingly important. Global demand for plant-based protein is rising rapidly, driven by population growth and shifting consumer preferences toward healthier and more sustainably sourced diets. The global plant protein market, valued at USD 29.4 billion in 2020, is projected to increase more than fivefold by 2030, potentially accounting for 7.7% of the total global protein market (Elkin, 2021). Legumes are central to this transition due to their low environmental footprint and high nutritional value, and they remain a primary source of dietary protein in many regions, particularly South Asia and the Middle East.

Among grain legumes, faba bean (*Vicia faba* L.) has emerged as a promising crop, containing two to three times more protein than cereals and higher protein levels than many other legumes, including peas, lentils, beans, and chickpeas (Chávez-Murillo et al., 2018; Rahate et al., 2021). Faba bean seeds typically contain 24–33% protein, along with carbohydrates, dietary fiber, minerals, bioactive compounds, and essential amino acids suitable for human and monogastric animal nutrition (Crépon et al., 2010; Koivunen et al., 2016; Valente et al., 2019; Khazaei et al., 2019). However, antinutritional compounds such as tannins, vicine, and convicine may limit utilization, particularly in non-ruminant diets. These compounds can be substantially reduced or eliminated through targeted breeding and processing approaches (Cabrera and Martín, 1986; Luo and Xie, 2013; Khazaei et al., 2017; Sharan et al., 2021).

Despite recent increases in global faba bean production, supply remains insufficient to meet growing demand. In 2024, global imports reached approximately 1 million tons of dry seeds and 0.06 million tons of green seeds (FAOSTAT, 2026). Closing this demand–supply gap requires accelerating yield gains and narrowing yield gaps. Globally, average dry faba bean yield increased from less than 1 t ha^−^¹ in 1961 to approximately 2.2 t ha^−^¹ in 2024, corresponding to an annual increase of about 1.28% (FAOSTAT, 2026). This yield improvement has also resulted in increased protein yield, rising from approximately 250 kg ha^−^¹ to nearly 550 kg ha^−^¹, underscoring the crop’s growing contribution to global plant protein production.

Improving grain yield and protein concentration simultaneously remains a key objective of faba bean breeding programs. Grain yield is a complex trait strongly influenced by environmental conditions and genotype × environment (G×E) interactions. Previous studies examining the relationship between grain yield and protein content in faba bean have reported inconsistent results, ranging from no clear association (Picard, 1977) to significant negative (Lizarazo et al., 2015; Barłóg et al., 2019) or positive correlations (El-Sherbeeny and Robertson, 1992; Skovbjerg et al., 2020). These contrasting findings highlight the complex and context-dependent nature of protein–yield relationships, emphasizing the need for advanced populations and multi-environment evaluations.

Biparental populations have been widely used in faba bean to identify QTLs associated with biotic and abiotic stress tolerance and key agronomic traits (Román et al., 2003; Arbaoui et al., 2008; Cruz-Izquierdo et al., 2012; Catt et al., 2017). However, their limited genetic diversity and restricted recombination reduce mapping resolution and statistical power for dissecting complex, polygenic traits such as grain yield and protein content. These limitations can be effectively addressed using Multi-parent Advanced Generation Inter-Cross (MAGIC) populations, which are developed by intercrossing multiple genetically diverse founder lines, resulting in high levels of recombination and a broad genetic base (Cavanagh et al., 2008; Huang et al., 2015; Scott et al., 2020). MAGIC populations have proven particularly powerful for resolving the genetic architecture of complex traits and for identifying loci with small to moderate effects across diverse environments. In faba bean, MAGIC populations have been successfully used to dissect traits including frost tolerance, flowering time, seed size, disease resistance, and yield-related traits (Sallam and Martsch, 2015; Khazaei et al., 2018; Skovbjerg et al., 2023). Notably, an eight-parent MAGIC population developed at ICARDA integrates diverse genetic backgrounds spanning the *paucijuga*, *minor*, and *equina* types, with substantial variation in protein content, seed size, grain yield, and phenological traits (Maalouf et al., 2019). This population provides a robust platform for simultaneously evaluating grain yield, protein concentration, and protein yield, as well as their interactions across environments, thereby supporting the development of high-performing faba bean cultivars for sustainable plant-based protein production.

This study exploits one of the largest faba bean MAGIC populations reported to date, developed through two cycles of eight-way intercrossing, enabling high recombination and broad genetic diversity. Unlike previous faba bean MAGIC studies that primarily focused on yield components or genetic mapping under limited conditions, this work integrates multi-environment field evaluation with high-throughput protein phenotyping to jointly dissect grain yield and protein content. The objectives of the present study were to evaluate this MAGIC population for grain yield and protein content, assess the feasibility of selecting lines combining high yield and protein content, and examine the relationships between protein content and other key agronomic traits.

## 2 Materials and methods

### 2.1 Plant materials

A total of 2,431 F6 MAGIC lines were developed through two rounds of eight-way F1 inter-crossing of 28 crosses for diverse biotic, abiotic, and quality traits, as indicated in **Table 1**.

**Table 1:** Pedigree and origin of parents involved in MAGIC population development.

Parent	Pedigree	Subspecies	Origin	Traits
Ascot	ILB1593	*Equina*	Germany (DEU)	Ascochyta Blight Resistance, released in Australia
Eddamer	Bulk selection-2009	*Equina*	Sudan	Heat tolerance, short duration, released in Sudan
Najah	Sel.88Lat.18025 X SP49C	*Minor*	ICARDA (Egypt x Spain) x Tunisian landrace	High yield, long duration, released in Tunisia
Misr3	Line 667 X (Cairo 241 X Giza 461):	*Equina*	ICARDA X Egypt	High yield, short duration
ICARUS	BPL710	*Equina*	Ecuador	Long duration, low water requirement
VF136	Selection from F402 x Alameda	*Minor*	Egypt x Spain	Highly auto fertile
Lattakia 1	Sel.99 lat.10268–3 selection from L 83 129	*Equina*	ICARDA -Lebanon	Short duration, with multiple disease resistance
NA112	Selection from ILB332	*Paucijuga*	Pakistan	Drought tolerant, highly auto fertile

### 2.2 Field experiments

**Figure 1:**
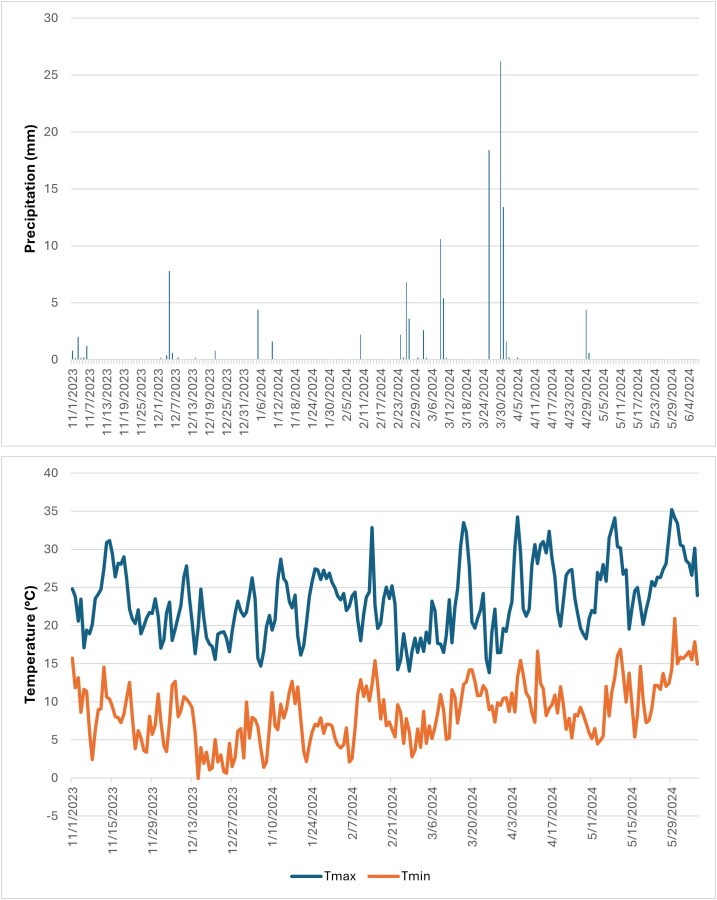
Precipitation (mm) and maximum and minimum air temperatures (°C) in Marchouch during 2023–2024 growing season.

**Figure 2:**
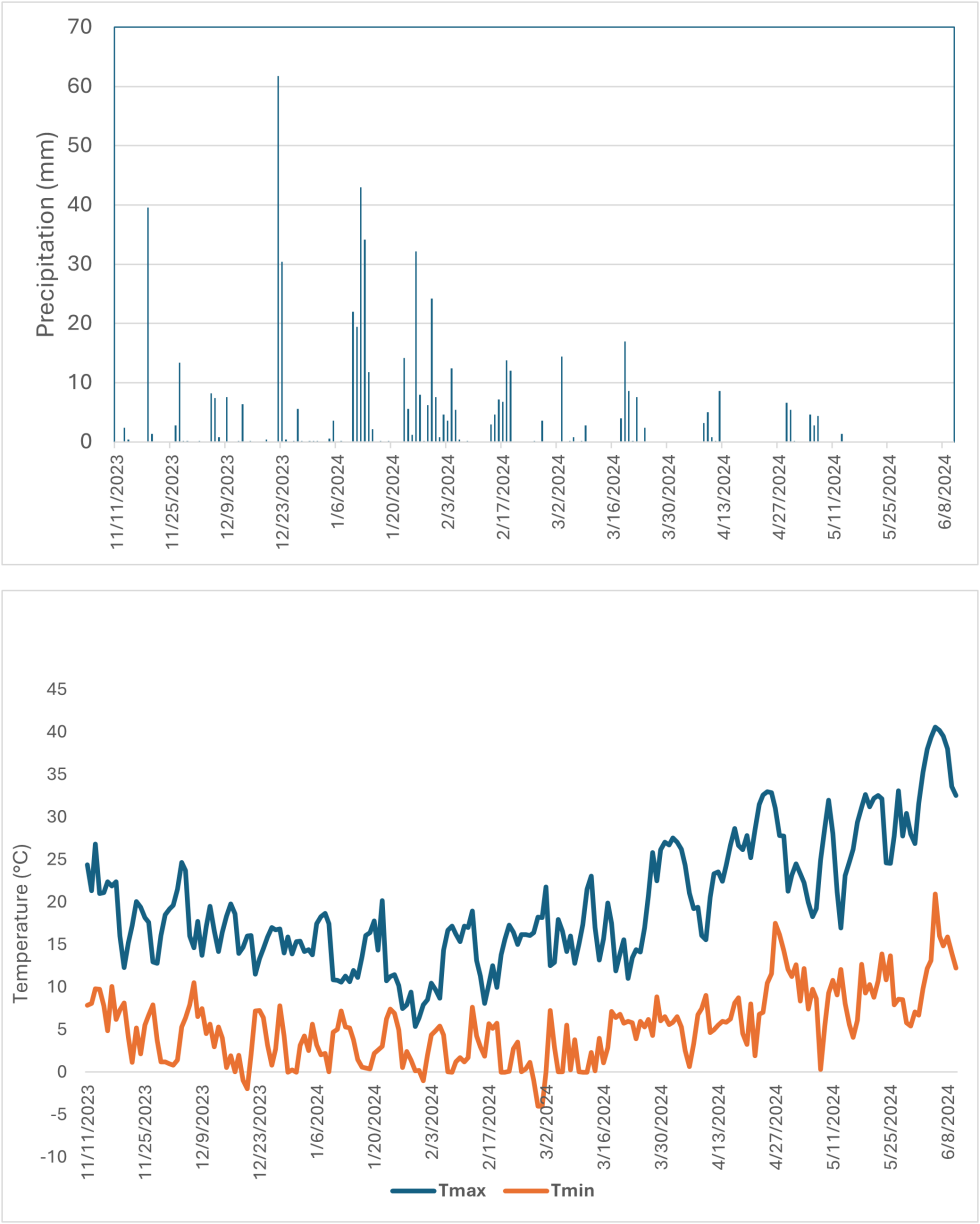
Precipitation (mm) and maximum and minimum air temperatures (°C) in Terbol during 2023–2024 growing season.

Two experiments were conducted at ICARDA research stations located in two different regions: one in Terbol (35.98° N, 33.88° E, 890 m asl) located in the Bekaa Valley of Lebanon, and the other in Marchouch (33.5581° N 6.6930°W, 255 m asl) in Morocco. Terbol station is characterized by deep and rich clay loam soil, cool and high rainfall winter and a moderate, wet spring. Marchouch station is characterized by vertisol and a mostly silty clay soil in a semiarid environment. In Terbol, the crop received a total precipitation of 607.8mm throughout the cropping season, with air temperatures ranging from a maximum of 40.6°C to a minimum of -4°C. The experiments were sown in late November at Terbol and mid-December at Marchouch and harvested by the end of May at both locations. The experiments were supplied with 250 kg/ha of granulated NPK (15:15:15) during land preparation and necessary phytosanitary and agronomic management practices were applied to ensure a good crop stand. In Marchouch, the crop received 120mm of precipitation, and temperatures varied from a maximum of 35.22°C to a minimum of -0.06°C during the cropping season. Patterns of precipitation and air temperature are presented in **Figures 1**, **2**. Supplemental irrigation with 30 mm was provided in Marchouch for seed establishment due to delayed rainfall.

The tested lines were planted using an augmented row-column design, along with their eight parents and four replicated checks (Najah, Misr-1, ICARUS and ASCOT). Each of the four checks was included in each of the 13 plots. The design comprised 264 blocks, resulting in a total of 3,432 plots. Each plot consisted of a single 2-m row spaced 45 cm apart, corresponding to a plot area of 0.90 m², with ten seeds sown per row.

### 2.3 Phenotypic data

The following data were recorded at both the locations: number of days from sowing to 50% flowering (DFLR_S), number of days from germination to 50% flowering (DFLR_G), number of days from sowing to 50% maturity (DMAT_S), number of days from germination to maturity (DMAT_G). In addition, the following data were recorded as an average of three random plants per plot: number of pods per plant (NPPLT), number of seeds per pod (NSP), number of seeds per plant (NSPLT), plant height (PLHT), biological yield per plant (BYPLT), and grain yield per plant (GYPLT), hundred seed weight (HSW).

### 2.4 Protein analysis

#### 2.4.1 Kjeldahl method

One hundred twenty faba bean samples were randomly collected from the field experiment of the MAGIC population at Marchouch and analyzed using the Kjeldahl method (Baethgen and Alley, 1989). Ground seed samples were digested by heating in a digestion block at 300°C for five hours in the presence of sulfuric acid, selenium, and salicylic acid. The digest was then treated with 5.5 mL of buffer solution, 4 mL of sodium nitroprusside, and 2 mL of sodium hypochlorite and incubated in the dark at 37°C for 15 minutes. Nitrogen content was multiplied by 6.25 to convert it to protein content estimates.

#### 2.4.2 Near infrared analysis

The tested MAGIC lines along with the checks were analyzed by non-destructive methods using FOSS NIR 2500 with its wavelength varying from 400 to 2,500 nm for samples collected at both Terbol and Marchouch stations. The faba bean protein content model was built using near-infrared spectroscopy (NIRS DS2500).

#### 2.4.3 Calibration model

A calibration model was developed based on grain samples. The effects of different mathematical and optical treatment methods on model performance were evaluated. The calibration was developed using Modified Partial Least Squares (MPLS) regression. The grain-based calibration validation results indicated that the best factors were the Standard Normal Variate (SNV)-detrend correction for optical treatment and the “2-2-2-2” combination for the mathematical method.

The model demonstrated an excellent predictive performance, achieving a Coefficient of Determination (R²) of 0.98. The Standard Error of Calibration (SEC) was 0.32, reflecting a low calibration error. The cross-validation technique of excluding v lines from the total evaluated lines, named Leave-v-Out Cross-Validation (LVN) yielded a Standard Error of Cross-Validation (SECV) of 0.75, while the correlation coefficient (1-VR) was 0.91. Additional validation was performed by splitting the dataset into training (70%) and test (30%) sets, achieving an R² of 0.82 for grain-based calibration.

### 2.5 Statistical methods

Quantitative data from each location were analyzed separately using the Automatic Restricted Maximum Likelihood (REML) method of row-by-column design of GenStat 23^rd^ edition. The fixed factor was Germplasm Identification Number (GID) while the random factors were row, column, block and replication.

For each trait, spatial covariance structures were formally evaluated using the GENSTAT automatic REML spatial procedure. Power-distance and separable row–column spatial models were tested, alongside alternative random-effect structures. The results of all REML model comparisons, including Deviance, AIC, and SIC values for spatial and non-spatial models, are presented in **Supplementary Table 1**. In each case, the model with the lowest AIC and SIC was retained. When spatial models did not improve fit or failed to converge, the GENSTAT automatic REML analysis of incomplete-block design was applied instead. Variations among lines were assessed in terms of *p-*values using the Wald statistic. Best linear phenotypic estimates (BLUPs) along with standard errors of differences were calculated for each line.

To evaluate the relationships among traits and classify the studied lines, several statistical analyses were performed. Correlation analysis between traits was conducted for each location separately to assess the relationship among phenological traits, yield traits and protein content. All quantitative traits were Z-score standardized prior to clustering. Hierarchical clustering was performed using Euclidean distance and Ward.D2 linkage. Cluster analysis grouped lines based on trait similarity using a 30% dissimilarity threshold at each location. To assess clustering quality, we calculated the cophenetic correlation coefficient (r = 0.32) and the mean silhouette width (0.07 for Terbol and 0.05 for Merchouch, respectively). Cluster stability was further evaluated using 500 bootstrap iterations with the pvclust package. The bootstrap dendrogram and silhouette plot are provided in the **Supplementary Figures**.

To identify traits contributing most to phenotypic variation, Principal Component Analysis (PCA) was performed on the standardized dataset. PCA biplots were generated to visualize the distribution of lines and their associations with key traits within the identified clusters. Additionally, a heat-map cluster analysis was produced for selected lines from both Terbol and Merchouch to compare protein content and yield performance relative to parental and check cultivars. Lines showing transgressive segregation with at least 25% higher protein content and grain yield than the average of the parental lines and check cultivars were identified.

## 3 Results

### 3.1 Agronomical traits

Differences among lines were highly significant (*p* < 0.001) for all evaluated traits, indicating wide phenotypic variation (**Table 2**). On average, time to flowering and maturity were longer at Terbol than at Marchouch. Flowering time ranged from 40 to 78 days after germination at Marchouch and from 60 to 94 days at Terbol, while maturity time varied from 90 to 128 days at Marchouch and from 136 to 167 days at Terbol.

**Table 2:** Wald statics, mean value & standard errors and minimum (Min) & maximum (Max) estimate per trait (***p<0.001).

Trait	Terbol					Marchouch				
	df	Wald Statistic	Means	Min	Max	df	Wald Statistic	Mean	Min	Max
DFLR_G	2436	7501.59***	75.6 ± 0.174	59.3	94.2	2438	8883.65***	44.2 ± 0.890	32.7	71.5
DFLR_S	2438	8316.35***	95.8 ± 0.590	86.9	116.0	2438	8883.65***	51.2 ± 0.890	39.6	78.5
DMAT_G	2436	4230.45***	150.4 ± 4.882	136.3	167.4	2437	6457.06***	108.3 ± 5.678	90.6	128
DMAT_S	2438	4422.09***	170.3 ± 0.65	157.6	185.3	2437	6457.06***	115.3 ± 5.678	97.6	135
PLHT	2437	3394.68***	80.4 ± 98.91	34.8	109.5	2438	3924.75***	52.0 ± 7.705	29.2	73.1
BYPLT	2408	3007.57***	98.9 ± 1.945	11	283.8	2401	2903.65***	24.7 ± 2.584	5.6	89.7
NPPLT	2435	2801.79***	21.1 ± 1.011	4.4	74.7	2419	3164.11***	8.06 ± 0.226	0.6	24.5
NSP	2419	4476.37***	3.52 ± 0.288	0.75	7.9	2422	3547.33***	2.58 ± 0.073	1.1	4.7
NSPLT	2427	4608.11***	69.8 ± 4.199	10.0	210.4	2428	4164.07 ***	20.7 ± 0.531	0.3	67.1
GYPLT	2435	2903.68***	42.5 ± 2.495	2.8	115.8	2417	2730.30***	11.3 ± 6.830	0.4	37.7
HSW	2429	14309***	63.5 ± 0.269	17.5	135.6	2423	6433.23 ***	56.1 ± 0.556	14.3	121.8
Protein_%	2436	11884.03***	25.5 ± 0.059	14	30.4	2400	4301.91***	24.3 ± 0.091	12.4	30.4

DFLR_G number of days from germination to 50% flowering; DFLR_S number of days from sowing to 50% flowering; DMAT_G number of days from germination to 50% maturity; DMAT_S number of days from sowing to 50% maturity; PLHT plant height; BYPLT biological yield per plant; NPP number of pods per plant; NSP number of seeds per pod; NSPLT number of seeds per plant; GYPLT grain yield per plant; HSW hundred seed weight; Protein_% percentage of protein content.

In addition, plant height, number of pods per plant, number of seeds per pod, number of seeds per plant, grain yield, biological yield, and protein content were consistently higher at Terbol than at Marchouch. Plant height ranged from 34.8 to 109.5 cm at Terbol and from 29.2 to 73.1 cm at Marchouch, while grain yield per plant varied from 2.8 to 115.8 g at Terbol and from 0.4 to 37.7 g at Marchouch. Protein content ranged from 14.0 to 30.4% at Terbol and from 12.4 to 30.4% at Marchouch (**Table 2**).

### 3.2 Relationship among the traits

**Figure 3:**
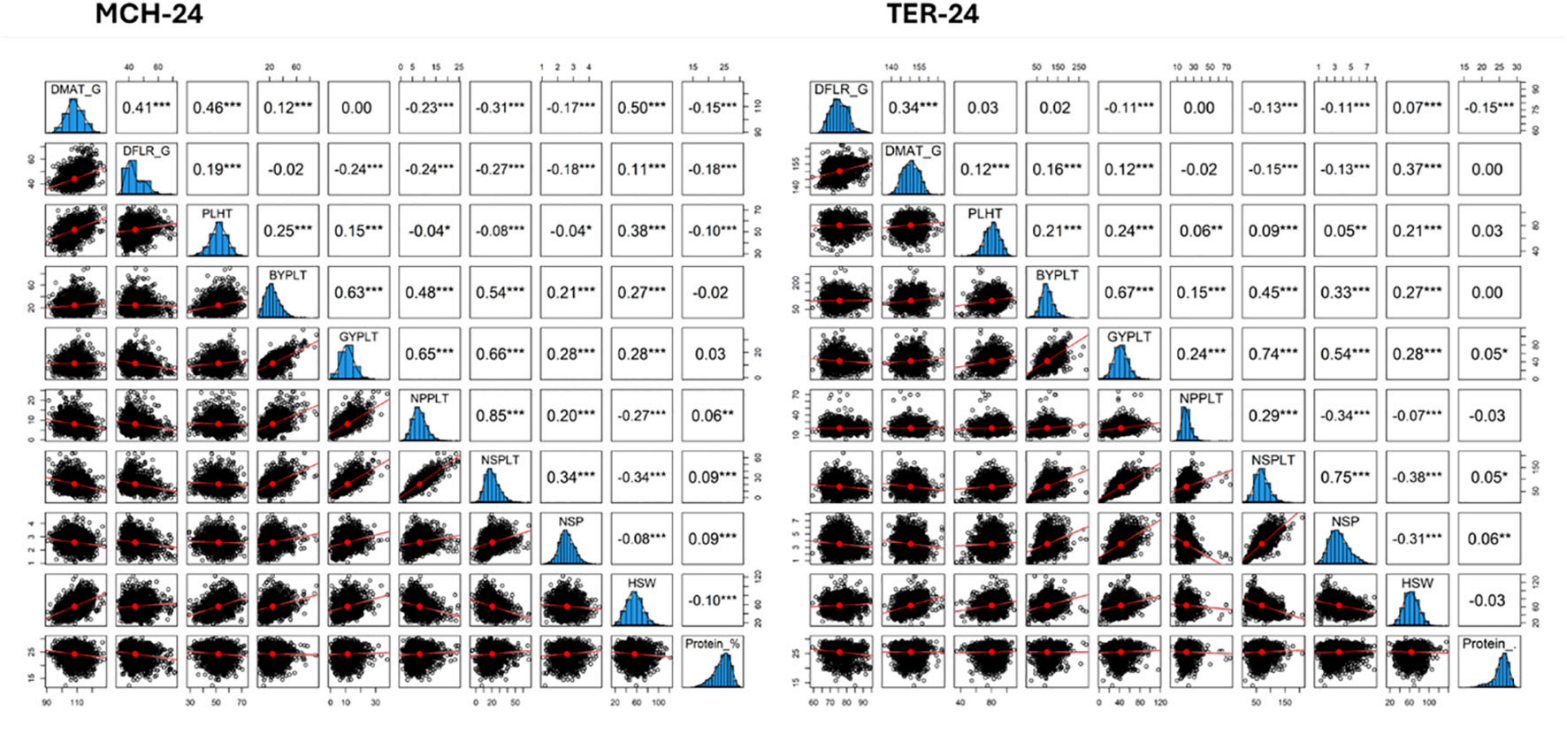
Correlation analysis of different traits evaluated in MAGIC population at Marchouch (MCH-24) and Terbol (TER-24). DFLR_G number of days from germination to 50% flowering; DFLR_S number of days from sowing to 50% flowering; DMAT_G number of days from germination to 50% maturity; DMAT_S number of days from sowing to 50% maturity; PLHT plant height; BYPLT biological yield per plant; NPP number of pods per plant; NSP number of seeds per pod; NSPLT number of seeds per plant; GYPLT grain yield per plant; HSW hundred seed weight; Protein_% percentage of protein content.

Correlation analysis of protein content across both locations was 0.6 (*p* < 0.001) indicating high replicability of protein estimates. Pearson’s correlation analysis was conducted to assess the relationships between the protein content, grain yield, yield attributes and phenological traits studied at Marchouch and Terbol in 2023/2024 (**Figure 3**).

Marchouch (Morocco) is characterized by a temperate climate and dry weather. At this site, protein content (Protein_%) was not correlated with grain (GYPLT) and biological yield per plant (BYPLT). Protein content showed low but significant positive correlations with number of pods per plant (NPPLT), number of seeds per plant (NSPLT), and number of seeds per pod (NSP). Selecting for high protein content was not strongly associated with yield and yield components, indicating the possibility to select faba bean with high protein content and high yield. In contrast, protein content exhibited significant negative but low correlations with hundred seed weight (HSW), indicating that larger seed size was generally associated with lower protein content. These observed low correlations provide opportunities to identify and select lines that can combine large seed size with high protein content and high yield, overcoming the trade-off for different customer preferences and benefits to growers. Additionally, significant negative correlations were observed between protein content and plant height (PLHT), as well as with the phenological traits, days to flowering and days to maturity. These correlations suggest that longer cycle durations and taller plants were associated with lower protein content.

At Terbol (Lebanon), characterized by a wet environment, protein content (Protein_%) showed significant positive correlations with plant height (PLHT), grain yield per plant (GYPLT), number of seeds per pod (NSP), and number of seeds per plant (NSPLT). This suggests that it is possible to select for all these traits simultaneously and that in this target production environment, selection for high seed yield is less likely to compromise high protein content selection in faba bean. Conversely, a negative but significant correlation was observed between protein content and days to flowering (DFLR), number of pods per plant (NPPLT), and hundred seed weight (HSW), suggesting that longer vegetative phase, higher pod number, and larger seed size are associated with lower protein content at this site.

### 3.3 Clustering the MAGIC lines based on agronomical traits and protein content

**Figure 4:**
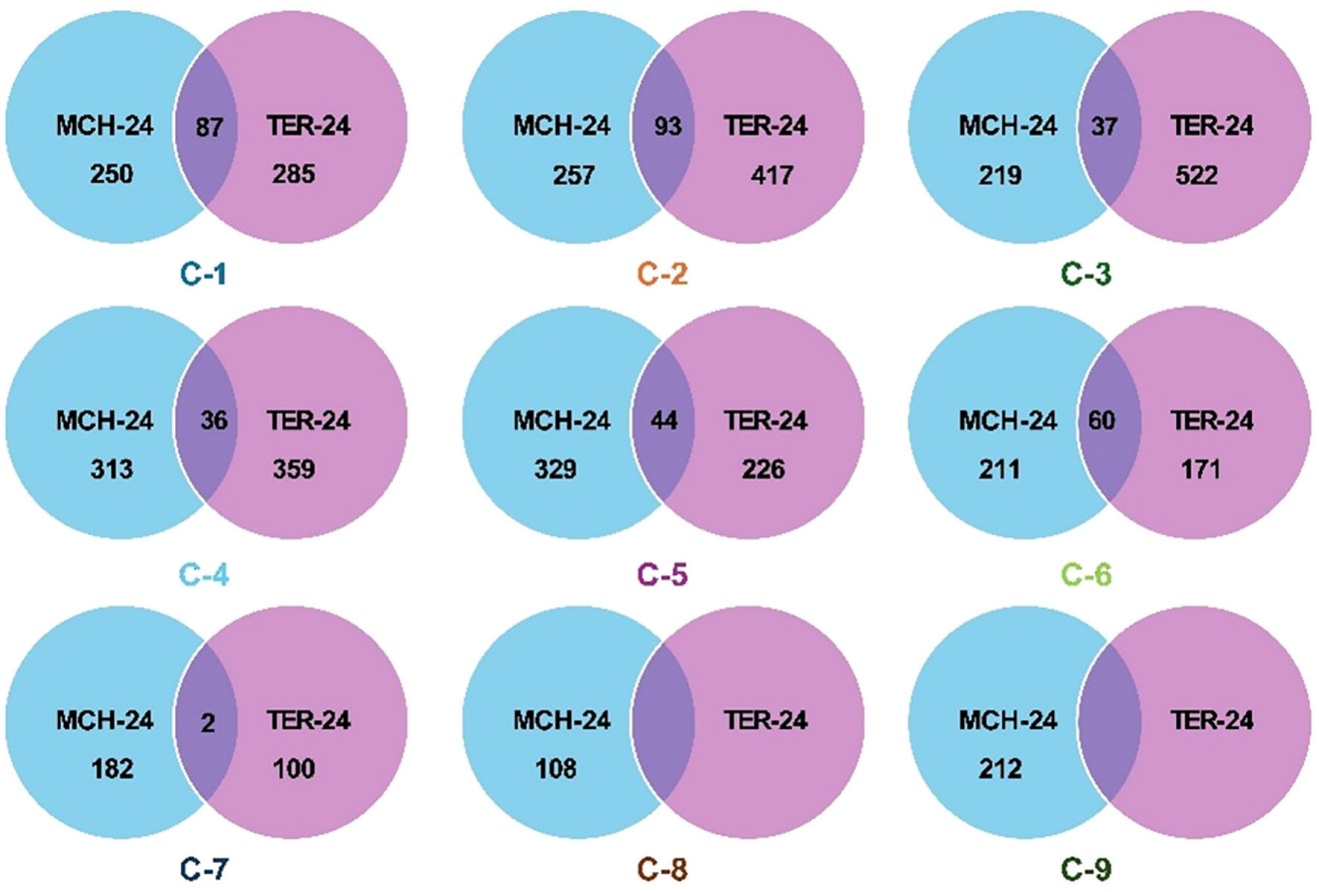
Distribution of MAGIC lines into trait-based clusters at Marchouch (MCH-24) and Terbol (TER-24).

Using cluster analysis, the MAGIC lines were classified based on their trait profiles into nine clusters (C1 to C9) at Marchouch and seven clusters at Terbol (C1 to C7) (**Figure 4**), with 70% similarity. The Venn diagrams illustrate the degree of overlap between the two locations, reflecting both shared and unique MAGIC lines within each cluster. The extent of similarity varied considerably, with the number of shared lines ranging from only two in C7 to as many as 93 in C2. Clusters with higher overlaps, such as C1, C2, and C6, suggest a greater consistency in trait-based grouping across environments, while clusters with minimal overlaps, including C7, C8, and C9, highlight site-specific divergence and potential environmental influence on trait expression.

Results in **Table 3** showed that at Marchouch, C7 exhibited the highest mean protein content (25.9%), followed by C4, C3, C8 and C2; which all had similar mean protein content. The remaining clusters include C5, C1, C9, with C6 were observed to have the lowest mean protein content (21.1%). Cluster 6 also had the widest range of protein content, varying from 12.2 to 27.9% and the lowest protein content (12.2%). Cluster 8 had the narrowest range, between 20.1 and 29.2%. MAGIC lines with the highest protein content (30.4) were reported to be in C4.

**Table 3:** Minimum, maximum and mean values of best linear unbiased estimates of traits for the different clusters in Marchouch 2024.

Cluster_MCH24		DMAT_G (days)	DFLR_G (days)	PLHT (cm)	BYPLT (g/plant)	GYPLT (g/plant)	NPPLT	NSPLT	NSP	HSW (g)	Protein _%
C1	Minimum	98.6	35.2	41.8	12.1	4.9	4.5	5.2	1.7	30.9	14.4
	Maximum	126.9	61.5	73.1	67.8	27.8	17.6	40.1	3.8	121.8	28.6
	Mean	112.6	45.3	56.6	33.4	14.6	9.2	23.0	2.6	66.0	23.5
C2	Minimum	95.4	33.8	35.3	12.1	5.5	3.0	10.1	1.8	24.6	19.9
	Maximum	115.3	50.5	64.1	51.3	22.0	14.7	46.9	4.7	86.4	29.9
	Mean	106.8	40.7	51.2	27.5	14.1	8.7	23.5	2.9	60.2	25.1
C3	Minimum	91.4	34.0	34.1	8.1	3.9	6.2	18.3	1.7	20.3	17.7
	Maximum	115.0	53.4	64.7	56.6	25.7	24.0	66.3	4.2	61.8	29.7
	Mean	103.4	41.8	50.8	29.8	13.2	12.2	34.3	2.7	41.3	25.2
C4	Minimum	103.1	33.1	39.3	5.9	0.5	0.6	-4.9	1.3	27.7	20.1
	Maximum	124.5	59.0	71.1	50.9	14.1	10.5	28.6	3.5	114.9	30.4
	Mean	111.9	45.9	54.5	21.4	8.7	5.8	13.7	2.4	61.4	25.5
C5	Minimum	94.2	33.2	32.2	5.6	0.5	1.8	0.7	1.2	17.5	17.8
	Maximum	115.9	67.9	62.2	50.9	16.0	13.5	34.9	3.7	74.3	30.0
	Mean	105.4	43.1	49.2	14.8	7.9	6.5	15.4	2.4	46.8	25.1
C6	Minimum	97.5	34.8	30.1	5.9	0.8	2.3	0.3	1.1	25.3	12.2
	Maximum	118.5	58.8	63.8	41.6	19.4	15.4	32.7	4.3	91.0	27.9
	Mean	108.4	44.4	49.3	19.5	9.7	7.0	17.3	2.5	55.8	21.1
C7	Minimum	90.6	33.1	29.2	5.9	1.2	2.6	6.0	1.7	19.4	21.0
	Maximum	111.2	48.0	58.3	36.4	14.7	15.3	42.8	4.4	70.0	30.1
	Mean	101.8	40.0	42.6	17.2	9.3	7.8	21.2	2.9	42.0	25.9
C8	Minimum	96.0	32.7	35.4	23.6	6.4	7.5	25.8	1.7	22.6	20.1
	Maximum	118.6	60.4	71.5	89.7	37.7	24.5	67.1	4.1	101.1	29.2
	Mean	107.4	42.4	54.9	46.2	20.8	13.6	37.7	2.8	58.3	25.1
C9	Minimum	102.4	39.6	43.8	5.6	0.1	0.3	-3.5	1.1	27.5	15.7
	Maximum	128.0	71.5	72.1	57.4	18.9	14.1	23.0	3.6	109.4	27.8
	Mean	115.5	54.3	58.0	23.5	8.7	5.4	12.0	2.3	70.8	21.8

DFLR_G number of days from germination to 50% flowering; DFLR_S number of days from sowing to 50% flowering; DMAT_G number of days from germination to 50% maturity; DMAT_S number of days from sowing to 50% maturity; PLHT plant height; BYPLT biological yield per plant; NPP number of pods per plant; NSP number of seeds per pod; NSPLT number of seeds per plant; GYPLT grain yield per plant; HSW hundred seed weight; Protein_% percentage of protein content.

The ranking of clusters from highest to lowest mean grain yield per plant were as follows: C8, C1, C2, C3, C6, C7, C9, C4, and C5. The mean values ranged from 7.9 in C5 to 20.8 in C8. Cluster 8 also showed the widest range of grain yield per plant, varying from 37.7 to 6.4, while C4 has the narrowest range, varying from 0.5 to 14.1. The highest yielding lines was reported in C8, while the lowest yield performance was in C9. In summary, at Marchouch, C8 was observed to have superior protein content and grain yield, while C6 was observed to have low performance for both traits.

**Table 4** showed that at Terbol, the seven clusters were ranked as follows in terms of protein content: C3 exhibiting the highest mean protein content (26.3%), followed by C1, C7, C2, C4, C5, and Cluster 6 with the lowest mean protein content (21.2%). Cluster 6 also has the widest range of protein content, varying from 14.0 to 25.5%, while C1 has the narrowest range, with values between 21.9 and 29.6%. MAGIC lines with the highest protein content belonged to C3, while the lowest protein content (14.0%) was reported to be in C6.

**Table 4:** Minimum, maximum and mean values of best linear unbiased estimates for traits of the different clusters obtained at Terbol 2024.

Cluster _TER24		DFLR_G (days)	DMAT_G (days)	PLHT (cm)	BYPLT (g/plant)	GYPLT (g/plant)	NPPLT	NSPLT	NSP	HSW (g)	Protein _%
C1	Minimum	63.7	142.4	55.6	40.0	22.4	9.2	20.9	1.5	33.3	21.9
	Maximum	93.2	164.3	106.5	225.0	74.9	47.2	127.7	5.8	135.6	29.6
	Mean	76.6	153.3	85.3	124.5	53.9	22.6	74.9	3.5	75.7	26.3
C2	Minimum	61.4	138.5	50.0	58.6	18.2	11.0	29.2	1.3	28.1	20.5
	Maximum	88.3	158.6	101.2	283.8	81.8	74.7	164.1	6.1	89.8	29.4
	Mean	72.4	148.1	81.0	100.5	43.0	24.2	75.9	3.4	58.3	26.0
C3	Minimum	66.3	144.4	55.1	31.0	9.5	8.9	16.1	0.8	17.5	21.1
	Maximum	92.8	167.0	105.0	181.7	73.3	36.4	105.5	6.5	98.3	30.4
	Mean	78.9	153.1	79.4	90.8	37.2	19.6	59.0	3.2	65.9	26.3
C4	Minimum	59.3	136.3	34.8	11.0	2.8	4.4	9.9	0.8	18.6	20.6
	Maximum	87.4	159.6	101.5	141.7	49.1	37.0	115.2	6.2	103.7	29.1
	Mean	73.6	147.5	73.9	65.4	26.0	19.1	50.5	2.8	54.9	25.8
C5	Minimum	66.2	138.6	60.5	55.0	28.3	11.2	59.3	3.7	29.3	18.5
	Maximum	87.5	156.0	109.5	182.0	82.4	33.7	177.7	7.9	84.5	28.9
	Mean	74.7	148.5	83.1	107.5	51.8	18.9	98.0	5.3	54.8	25.3
C6	Minimum	65.8	140.1	53.0	24.0	7.8	9.7	12.1	1.3	36.5	14.0
	Maximum	94.2	164.5	101.2	195.7	68.3	38.2	95.4	5.4	116.8	25.5
	Mean	78.9	151.3	80.6	96.7	39.1	21.2	55.2	2.8	73.2	21.2
C7	Minimum	65.2	143.1	62.8	92.1	49.8	10.7	70.9	3.4	39.8	19.1
	Maximum	84.8	167.4	106.4	270.0	115.8	33.2	210.4	7.9	91.0	28.9
	Mean	73.8	152.1	83.9	154.0	75.0	21.5	113.2	5.5	67.3	26.1

DFLR_G number of days from germination to 50% flowering; DFLR_S number of days from sowing to 50% flowering; DMAT_G number of days from germination to 50% maturity; DMAT_S number of days from sowing to 50% maturity; PLHT plant height; BYPLT biological yield per plant; NPP number of pods per plant; NSP number of seeds per pod; NSPLT number of seeds per plant; GYPLT grain yield per plant; HSW hundred seed weight; Protein_% percentage of protein content.

Ranking clusters from highest to lowest, the mean grain yield per plant was as follows: C7 with the highest at 75.0, followed by C1, C5, C6, C3, and finally C4 with the lowest mean grain yield per plant value equal to 26.0. Cluster 7 showed the widest range of grain yield per plant, varying from 49.8 to 115.8, while C4 had the narrowest range, varying from 2.8 to 49.1. Cluster 7 included lines having the highest grain yield per plant (115.8), while C4 exhibited the lowest grain yield per plant (2.78). In summary, at Terbol, C7 and C1 were reported to have superior protein content and grain yield, while C6 showed low responses in both traits.

### 3.4 Major traits and their relationship with protein

**Figure 5:**
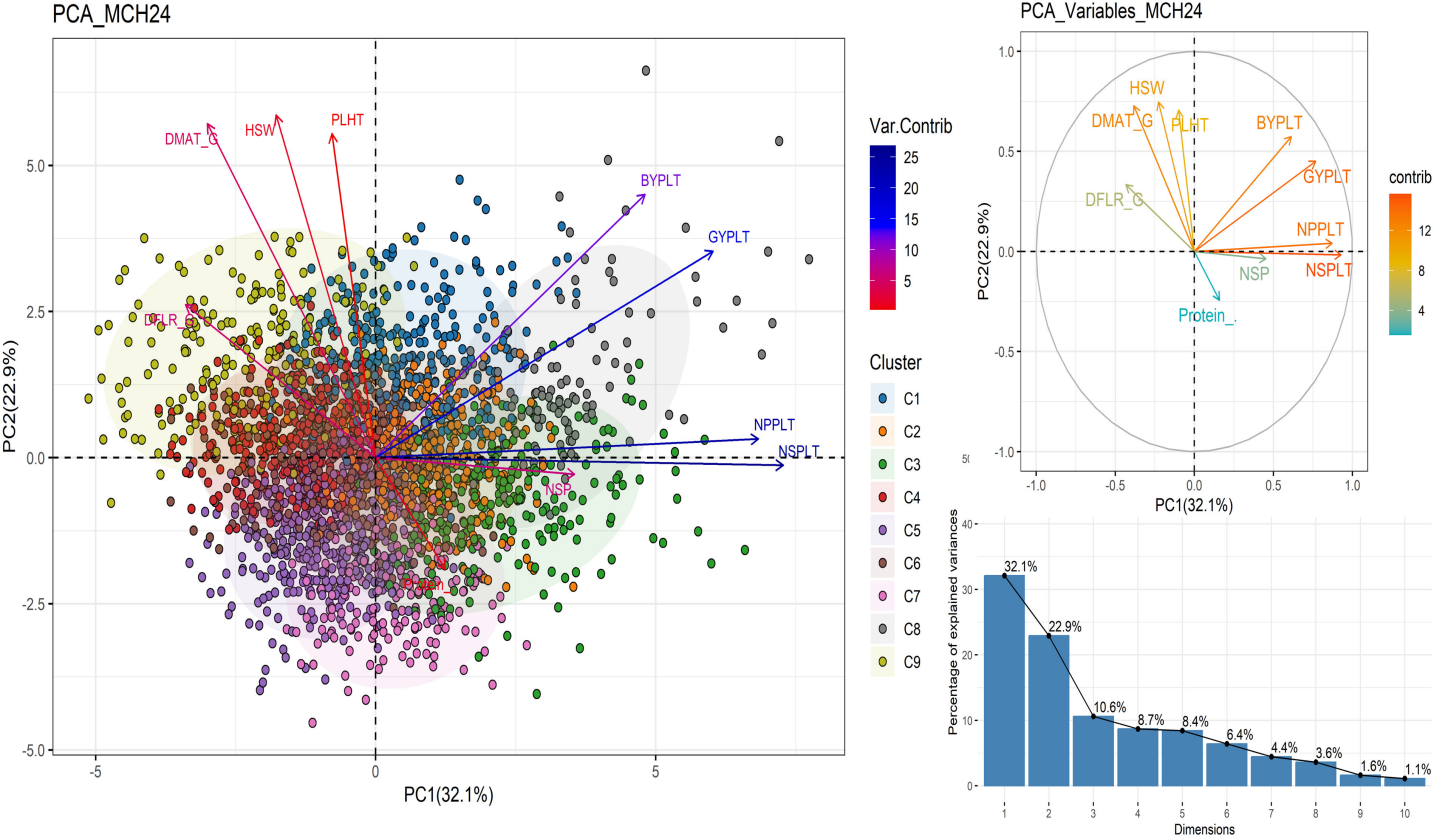
Principal components analysis using best unbiased genotypes generated at Marchouch station. DFLR_G number of days from germination to 50% flowering; DFLR_S number of days from sowing to 50% flowering; DMAT_G number of days from germination to 50% maturity; DMAT_S number of days from sowing to 50% maturity; PLHT plant height; BYPLT biological yield per plant; NPP number of pods per plant; NSP number of seeds per pod; NSPLT number of seeds per plant; GYPLT grain yield per plant; HSW hundred seed weight; Protein_% percentage of protein content.

The PCA revealed that the first three principal component dimensions (PC1, PC2, PC3) together explained 65.6% of the total variance at Marchouch (**Figure 5**; **Table 5**). Cluster groups C1 to C9 were also presented in different colors. NSPLT and NPPLT vectors are long and dark blue, indicating that these traits account for the largest portion of variance, followed by GYPLT, BYPLT, NSP, DMAT, DFLR, PLHT, HSW, and protein content, respectively. The protein content vector is red and short, reflecting its minimal contribution to the overall variance. PC1 explained a 32.1% variation and is highly associated with the number of seeds and pods per plant. The PC2 explained the 22.9% variation is associated with GYPLT, PLHT, DMAT_G and HSW. PC3 explained that the 10.6% variation is positively and highly associated with protein content and moderately associated with HSW (**Table 5**).

**Table 5:** Correlation between the three main principal component axes and the study’s studied traits at Terbol and Marchouch.

Traits	Marchouch			Terbol		
	PC1(32.1%)	PC2(22.9%)	PC3(10.6%)	PC1(28.4%)	PC2(19.5%)	PC3(12.5%)
DMAT_G	-0.38	0.73	-0.03	-0.16	0.40	-0.14
DFLR_G	-0.43	0.33	-0.51	-0.04	0.70	-0.22
PLHT	-0.10	0.71	0.05	0.26	0.40	0.01
BYPLT	0.61	0.57	-0.03	0.71	0.41	0.01
GYPLT	0.77	0.45	0.11	0.90	0.28	0.05
NPPLT	0.87	0.04	-0.26	0.19	0.13	0.94
NSPLT	0.93	-0.02	-0.21	0.91	-0.28	0.13
NSP	0.45	-0.04	0.28	0.75	-0.36	-0.50
HSW	-0.23	0.75	0.40	-0.07	0.81	-0.11
Protein_%	0.16	-0.24	0.66	0.09	-0.11	-0.11

MAGIC lines located on the lower-right side of the plot exhibited the highest protein content, while those on the upper-right side have the highest yield. Lines positioned between the GYPLT and Protein_% vectors performed well for both traits and for the BYPLT, and the grain yield components NPPLT, NSPLT and NSP, as all their vectors were positioned in the same direction. However, **Figure 5** demonstrates that these lines also were reported to have low plant height, small seed size, and a short crop cycle. These lines were predominantly from clusters C2, C3, and C8, with a minority from all other clusters, excluding C9.

**Figure 6:**
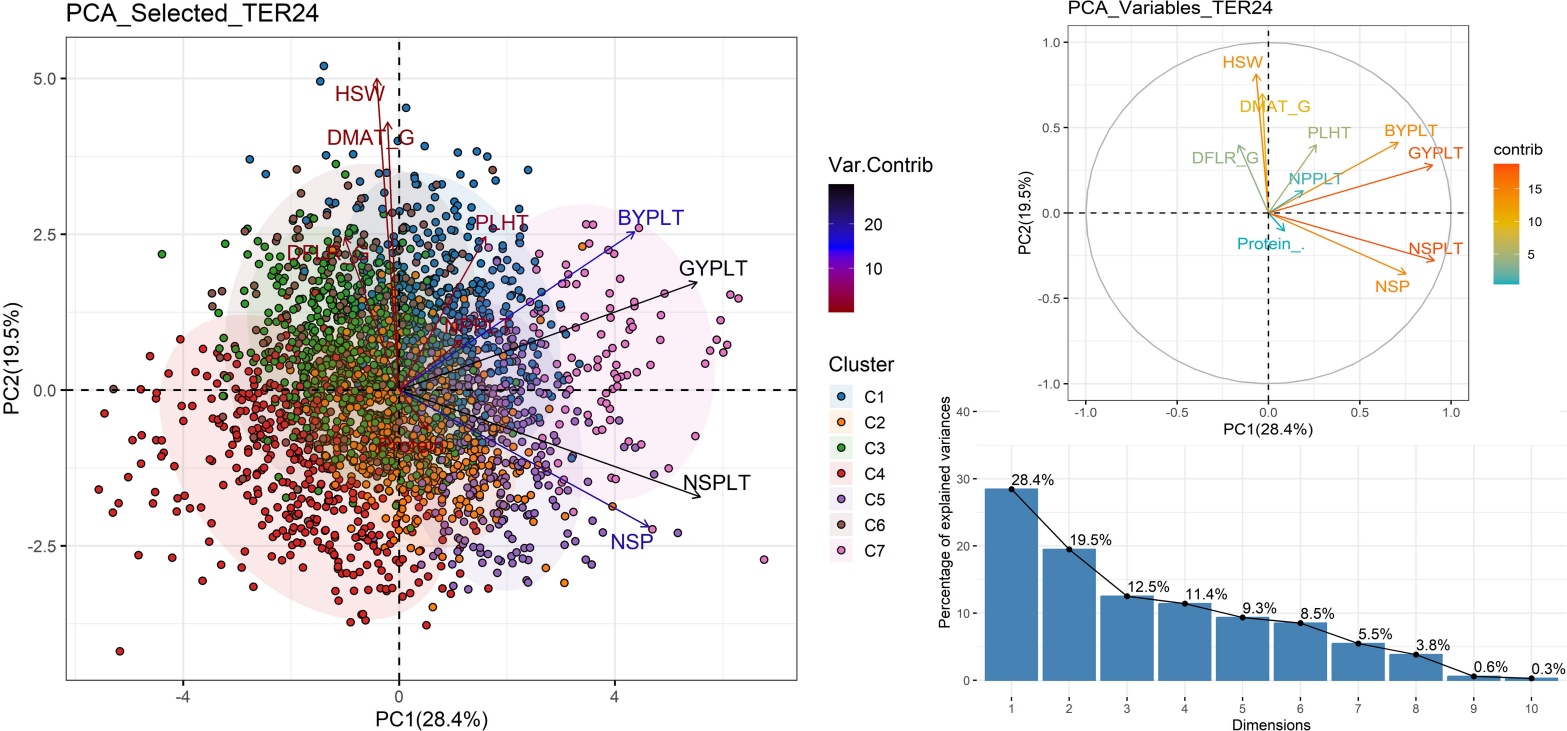
Principal components analysis using best unbiased genotypes generated at Terbol station. DFLR_G number of days from germination to 50% flowering; DFLR_S number of days from sowing to 50% flowering; DMAT_G number of days from germination to 50% maturity; DMAT_S number of days from sowing to 50% maturity; PLHT plant height; BYPLT biological yield per plant; NPP number of pods per plant; NSP number of seeds per pod; NSPLT number of seeds per plant; GYPLT grain yield per plant; HSW hundred seed weight; Protein_% percentage of protein content.

The PCA analysis revealed that the first three dimensions together explained 60.4% of the total variance at Terbol (**Figure 6**, **Table 5**). The clusters described previously for Terbol were also visualized with different colors in **Figure 6**. The first PC1 explained 28.4% and was associated with NSPLT, NSP, and GYPLT. PC2 explained 19.5% of total variation and was associated with HSW and DFLR highlighting the importance of seed size and cycle duration in explaining the variance captured by this component. PC3 explained 12.5% and was associated with NPPLT indicating that this dimension primarily reflects variations in yield components.

**Figure 6** showed that the GYPLT, NSPLT, and NSP vectors were long and dark blue, indicating that these traits explain the largest portions of the variance, followed by PLHT, HSW, DMAT, DFLR, NPPLT, and protein content, respectively. Similar to Marchouch, the protein content vector was short and red, signifying its smaller contribution to the overall variance.

MAGIC lines on the lower-right side were reported to have high protein content, while those on the upper-right side were reported to have high yield. Lines situated between the GYPLT and Protein_% vectors on the right side showed high protein content with high grain yield. In addition, **Figure 6** shows that these lines performed well in terms of vegetative growth represented by BYPLT and PLHT and grain yield components (NPPLT, NSPLT, NSP) while having small seed size and a short crop cycle. Most of these lines belong to clusters C4 and C7, with a minority from other clusters, except for C5 and C6.

### 3.5 Selected MAGIC lines

**Figure 7:**
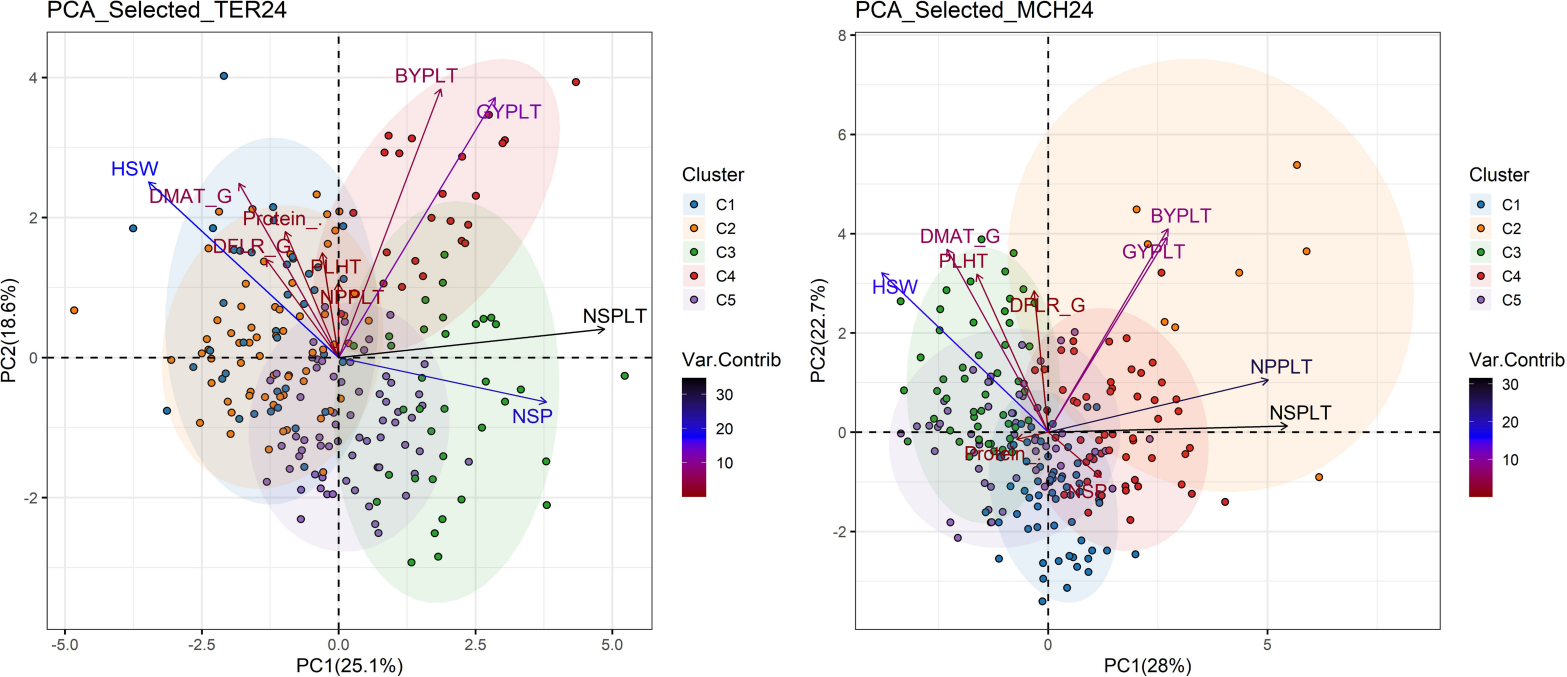
Principal component analysis using MAGIC lines selected for high grain yield and protein content performance in Terbol (TER24) and Marchouch (MCH24). DFLR_G number of days from germination to 50% flowering; DFLR_S number of days from sowing to 50% flowering; DMAT_G number of days from germination to 50% maturity; DMAT_S number of days from sowing to 50% maturity; PLHT plant height; BYPLT biological yield per plant; NPP number of pods per plant; NSP number of seeds per pod; NSPLT number of seeds per plant; GYPLT grain yield per plant; HSW hundred seed weight; Protein_% percentage of protein content.

MAGIC lines with superior grain yield and protein content compared to major cultivars in Australia (Ascot and Icarus) were selected at both Terbol and Marchouch. The PCA was reconducted to assess their performance in terms of other traits studied (**Figure 7**).

In the case of Marchouch, PC1 explained 28% of the total variation and was highly associated with number of seeds and pods per plant. PC2 explained 22% of variation and was positively associated with HSW, DMAT, DFLR, PLHT and BYPLT (**Figure 7**, PCA_Selected_MCH 2024).

**Figure 7** showed that at Marchouch, MAGIC lines were classified into five color-coded clusters. In addition to having high yield and high protein content, each cluster had groups of lines with common traits. For example, lines in C1 had high NSP, in C2 had good BYPLT, NSPLT and NPPLT, and C3 lines were tall (PLHT), large seeded (HSW), and had long crop cycles (DFLR, DMAT). MAGIC lines located in C4 had high yield components (NSP, NSPLT and NPPLT) and high BYPLT.

At Terbol, **Figure 7** shows that lines grouped in C1 and C2 were tall (PLHT) with large seed size (HSW), long cycle duration (DFLR, DMAT), and a large number of pods per plant. Cluster 3 lines had high grain yield and yield components (GYPLT, NSP, NSPLT). Cluster 4 contained lines with good biological grain yield, plant height and number of pods per plant.

### 3.6 Common selected lines

From Terbol and Marchouch trials, 403 MAGIC lines with high yield and high protein content were selected. Among them, 51 lines were ranked similar at both locations.

**Figure 8:**
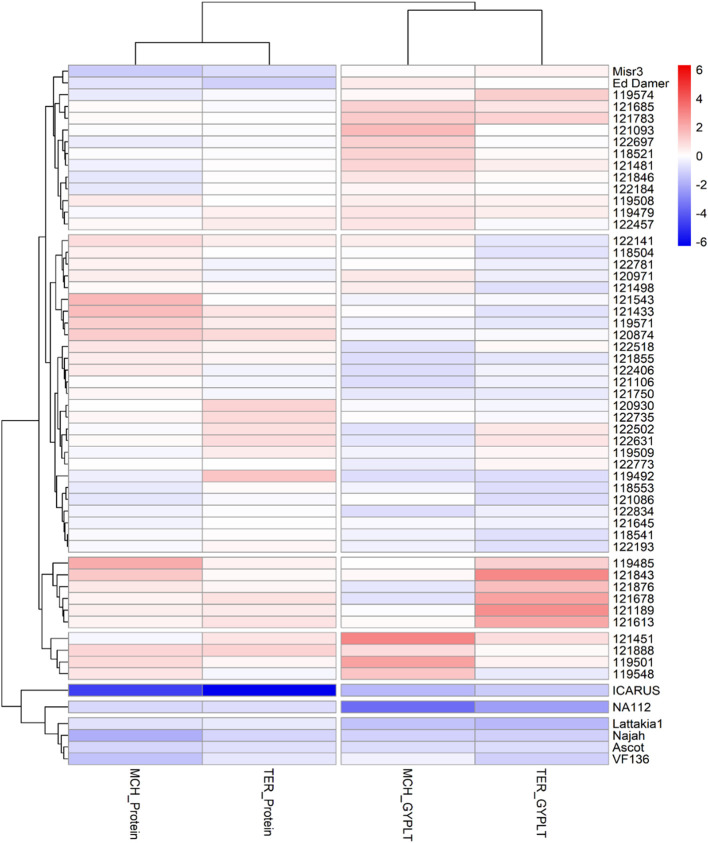
Heat map for common selected MAGIC lines at Terbol and Marchouch with higher grain yield per plant (g) and higher protein content (%) than the released cultivars (Ascot and Icarus in Australia).

A heat map (**Figure 8**) provided a visual representation of the selected lines, in addition to comparison with their parents and checks based on their performance, demonstrating superior yield and protein content. It classified the selected lines into seven clusters. The scale ranges from -6 to 6, highlighting the variability in trait performance among the 51 selected MAGIC lines.

Shades of blue highlighted lines with lower performance. For instance, NA112, Lattakia 1, Najah, VF136, in addition to the two Australian cultivars Icarus and Ascot (classified in the same cluster),consistently exhibited varying intensities of blue. This indicates suboptimal performance of the checks and parent comparisons when assessing protein content and grain yield and ranked lower compared to the 51 selected MAGIC lines across both locations (**Table 6**). Notably, the cultivar Icarus stands out with the darkest blue color for protein content at both locations, indicating the lowest performer for this trait. Misr 3 and Ed Damer have moderately high grain yield and moderately low protein content compared to the 51 selected lines.

**Table 6:** Germplasm identification number (GID) and pedigree of the top-performing MAGIC lines and checks across Marchouch and Terbol in terms of grain yield and protein content.

GID	Designation	Marchouch		Terbol	
		GYPLTg	Protein_%	GYPLTg	Protein_%
119485	Najah x Ascot/Misr3 x Ed Damer	15.4	29.7	67.6	28
119501	Najah x Ascot/Misr3 x Ed Damer	23.8	28.1	58.3	27.9
119548	Misr3 x Ed Damer/Najah x Ascot	20.5	27.7	48.3	27.3
121189	Misr3 x VF136/Najah x BPL710	15.1	27.5	86.6	28.2
121451	Najah x NA112/Misr3 x Lattakia1	26.3	26.5	64.1	28.4
121613	Misr3 x Lattakia1/Najah x NA112	15.8	27.2	79.1	28.5
121678	Najah x Ed Damer/BPL710 x Lattakia1	12.8	27.2	80.8	28.5
121843	BPL710 x Lattakia1/Najah x Ed Damer	16.0	28.7	88.3	27.8
121876	BPL710 x Lattakia1/Najah x Ed Damer	13.1	27.6	72.8	27.9
121888	BPL710 x Lattakia1/Najah x Ed Damer	18.5	28.3	55.8	29.0
Average of parents and Checks		12.3	24.6	42.6	25.7

Among the 51 selected lines, **Figure 8** showed that the cluster containing lines 119548, 119501, 121678 and 121451 had the highest protein content and highest yield among all selected lines. This was followed by clusters that contained lines 121613, 121189, 121678, 121876, 121843 and 121843. All ten lines emerged as the best performers (**Table 6**), consistently showing high protein content and grain yield at both locations. These 10 lines showed transgressive segregation with at least 25% higher protein content and grain yield than the average of the parental lines and check cultivars.

## 4 Discussion

This study evaluated protein content and a range of agronomic traits in MAGIC lines derived from an eight parental MAGIC population grown at two distinct locations. Significant differences among recombinant inbred lines (RILs) were expected, given that these lines were developed from the combination of eight genetically diverse parents representing different botanical groups of faba bean. Also, MAGIC populations are known for their high genetic diversity, which allows for a wide range of phenotypic traits to be expressed (Jiang and Xu, 2024).

Faba bean MAGIC lines flowered and matured earlier at Marchouch than at Terbol, due to higher temperatures and terminal drought conditions, which accelerated growth and development as part of a drought escape response. These stress conditions also negatively impacted growth, leading to reduced plant height and yield traits at Marchouch. The observed reductions are likely linked to impaired reproductive development, limited water uptake, and decreased photosynthetic efficiency under heat and drought conditions (Awasthi et al., 2014; Bishop et al., 2016; Abdelhaleim et al., 2022). Similar observations have been reported in faba bean (Maalouf et al., 2015; Siddiqui et al., 2015; Abou-Khater et al., 2022) and other legumes like chickpea (Awasthi et al., 2014), lentil (Sehgal et al., 2017; Choukri et al., 2020), and common bean (Beebe et al., 2013; Seidel et al., 2016).

The average protein content in MAGIC lines was slightly lower at Marchouch compared to Terbol. The lower protein content observed at Marchouch was likely due to impaired nitrogen fixation and reduced protein biosynthesis, which are typical responses to heat and drought that limit protein accumulation in seeds (Apel and Hirt, 2004; Kabbadj et al., 2017). However, due to the large number of accessions evaluated in this study, direct physiological measurements related to nitrogen fixation or assimilation were not feasible. Therefore, this interpretation should be regarded as a working hypothesis that requires future validation through targeted approaches such as on-site δ¹^5^N determination and post-anthesis nitrogen remobilization assessment. Fixed nitrogen supports both vegetative biomass accumulation and reproductive development, thereby influencing seed yield and grain protein content (Neugschwandtner et al., 2015). Similar reductions in seed protein content have been reported in lentil, chickpea and common bean under heat and combined heat and drought (Choukri et al., 2020; Behboudian et al., 2001; Ghanbari et al., 2015). While some studies have observed increases in certain stress related proteins under stress (Pareek et al., 1998), the overall reduction observed in our study supports the notion that environmental stress generally suppresses protein synthesis in faba bean. These differences are also likely influenced by genetic variation, as drought-tolerant varieties often continued to produce certain stress-related proteins, while susceptible ones showed greater reduction in protein content (Abdellatif et al., 2012; Belal et al., 2018; Hummel et al., 2018; Samineni et al., 2022). Protein content measured at both locations showed a moderately strong and statistically significant correlation. This indicates that, although the overall protein contents were lower at Marchouch, the ranking of the lines remained fairly consistent at both locations Therefore, genotypes that performed well at one location tended to perform well at the other, indicating reliable protein estimates across environments.

Understanding the relationship between protein content and other traits is crucial for developing breeding strategies that enhance protein content while maintaining yield and other important agronomic traits. Our findings highlight that environmental conditions influence how protein content correlates with other agronomic traits. Notably, the absence of a yield–protein trade-off observed in this study refers to the lack of a significant negative relationship between protein content and grain yield across environments, indicating that improvements in one trait do not necessarily reduce the other.

At Marchouch, protein content was not correlated with grain or biological yield. This suggests that both traits can be selected independently during breeding under similar environmental conditions, with improvements in one not necessarily limiting progress in the other. Previous studies have also reported an absence of correlation between protein content and seed yield in faba bean (Picard, 1977), as well as in lentil (Choukri et al., 2020) and in chickpea when evaluated under rainfed conditions in Southern Italy where the climate is similar to Marchouch (Sellami et al., 2021). On the other hand, positive correlations were observed between protein content and the number of pods and seeds per plant, indicating that greater pod and seed numbers are associated with high protein content. In contrast, other studies reported a negative correlation between these traits in many legume crops, such as lentil, pea and chickpea (Stoddard et al., 1993; Lizarazo et al., 2015; Gaur et al., 2016). Additionally, shorter plants with earlier flowering and maturity tended to have higher protein content, making them suitable targets for breeding in stress-prone environments. Although a negative correlation was found between protein content and seed size, the relationship was weak, suggesting that it may be possible to identify genotypes that combine both large seed size and high protein content. Lafiandra et al. (1981) also reported a negative correlation between faba bean protein content and seed size and Gaur et al. (2016) reported similar observations in chickpea.

In contrast, at Terbol, with its cooler and wetter climate, protein content showed positive correlations with plant height, grain yield, seeds per plant, and seeds per pod indicating that in favorable environments, it is possible to simultaneously improve yield and protein content. This might be attributed to efficient nitrogen fixation under favorable conditions, enabling plants to support these traits without the typical trade-off. Additionally, taller plants often have more efficient nutrient uptake, including nitrogen fixation, which contributes to protein synthesis. These findings are consistent with previous research (El-Sherbeeny and Robertson, 1992) that reported positive correlations between protein content and grain yield in faba bean and demonstrated that selecting for both yield and high protein content is achievable. Nevertheless, like Marchouch, protein content remained negatively correlated with pod number, seed size, and days to flowering, suggesting some consistent trade-offs at different environments. The absence of a yield–protein trade-off in this study likely reflects both genetic and physiological factors. Extensive recombination in the MAGIC population may have disrupted unfavorable linkages between loci controlling grain yield and protein content, allowing favorable alleles for both traits to be combined (Jiang and Xu, 2024). In addition, as a nitrogen-fixing legume, faba bean can support protein accumulation without directly competing with yield formation. Previous studies in faba bean and other legumes have similarly reported an absence of correlation between seed yield and protein content, attributed to the capacity for biological nitrogen fixation (Frimpong et al., 2009; Stoddard et al., 1993; Picard, 1977; El-Sherbeeny and Robertson, 1992). Cluster analysis revealed considerable genetic variability among the faba bean MAGIC lines, with nine clusters identified at Marchouch and seven at Terbol. These differences reflect both genotypic diversity and the influence of different environmental conditions. Furthermore, the observed variability among clusters also highlights the advantage of using multiparent lines for selection.

The ranking of clusters C7 and C1 at Terbol demonstrated that simultaneous improvement for protein content and grain yield is achievable, aligning with the positive and significant correlation observed between these two traits at this location. However, cluster C8 at Marchouch also showed strong performance for both traits, despite the absence of a correlation between protein content and grain yield in that environment. This likely reflects the use of multiparent lines in this study, as their broad genetic diversity increases the chance of identifying plants that combine multiple traits, like high yield and high protein content, making them a valuable resource for combined trait selection in breeding programs.

The PCA results visualized through biplot provide a comprehensive overview of the diversity among the MAGIC lines and their relationships with the studied traits. It also underscores the complexity of trait interactions in faba bean and their strong dependence on environmental conditions. At Marchouch, protein content contributed minimally to the total trait variation and showed a weak association with grain yield and its components as indicated by the nearly perpendicular vectors in the biplot. The observed trend in trait distribution confirms the absence of correlation between them in this environment.

In contrast, the PCA conducted at Terbol, a more favorable environment, revealed that although protein content had a minimal contribution to the total trait variation it showed stronger positive associations with the number of seeds and grain yield. While this association was not very strong, it suggests a higher likelihood of simultaneous improvements for protein content and grain yield in environments similar to Terbol. The closer alignment of protein content with traits like grain yield, seed number, and pod number supports the concept that under optimal conditions, plants can absorb and translocate nitrogen into the seed, improving both yield and seed quality.

Importantly, the lines exhibiting superior performance across multi-trait, multi-environment evaluations underscore the utility and robustness of MAGIC populations. The populations effectively combined diverse alleles to generate superior lines and enhance genetic gain. Their broad genetic diversity likely facilitates the identification of recombinant lines that integrate high protein content with favorable agronomic traits, even under environmental constraints. These lines represent valuable resources for breeding programs focused on the improvement of multiple traits and for investigating genotype-by-environment interactions underlying complex trait expressions. A total of 403 faba bean MAGIC lines exhibiting high protein content and grain yield relative to two Australian cultivars, Ascot and Icarus, were selected at each location. These lines will be re-evaluated in Australia in faba bean production areas. Re-clustering of the selected lines enhances the understanding of trait profiles, facilitates the identification of high-performing lines with shared agronomic characteristics, and enables assessment of the stability of trait-combinations across environments. This study also provides a valuable resource for targeted breeding efforts.

The selected lines exhibited a distinct clustering pattern compared to the full set, indicating differentiation in their trait profiles. At Marchouch, lines exhibited distinct trait profiles within clusters. MAGIC lines in C1 showed strong potential for maximizing seed yield. C2 was characterized by favorable performance in key yield components (BYPLT, NSPLT, NPPLT). C3 appeared to benefit from an extended vegetative period supporting biomass accumulation and seed filling; this group included tall lines with larger seeds and longer crop cycles. MAGIC lines in C4 combined high values for yield components with elevated biological yield per plant, suggesting the presence of highly productive recombinant lines carrying favorable alleles for both yield and biomass traits.

At Terbol, cluster analysis of the selected high-yielding and high-protein MAGIC lines revealed a distinct grouping pattern. C1 and C2 comprised of tall plants with large seeds, a higher number of pods, and extended growth periods, traits likely influenced by specific combinations of parental alleles. C3 is particularly valuable for selection and breeding programs targeting grain yield improvement in Terbol-type environments, as it includes MAGIC lines with high grain yield and yield components, potentially harboring novel combinations of yield-enhancing alleles generated through the MAGIC design. C4 contained lines with favorable yield, plant height, and pod number per plant, suggesting a balanced inheritance of alleles contributing to both biomass and yield-related traits under the environments assessed in this study.

The contrasting clustering patterns observed at Marchouch and Terbol highlight the significant influence of genotype-by-environment interactions on the performance of recombinant MAGIC lines. The diverse genetic backgrounds within the MAGIC population enable a wide spectrum of phenotypic responses across environments, highlighting the effectiveness of the intercrossing and selection strategy in generating novel and potentially superior genotypes.

The top performing 51 MAGIC lines across locations were selected for further analysis. Among these, 10 lines – 119548, 119501, 121678, 121451, 121613, 121189, 121678, 121876, 121843 and 121843 – consistently exhibited the highest protein content and grain yield. These lines emerged as the most promising, demonstrating stable performance across environments. Notably, they demonstrated transgressive segregation, outperforming both the parental lines and check cultivars, thereby highlighting the effectiveness of the MAGIC design in generating novel and favorable allele combinations and the unique value of this MAGIC population for protein-oriented faba bean breeding. Similar patterns of transgressive segregation have also been reported in eight-parent MAGIC populations of common bean and cowpea, supporting the broader applicability of this breeding strategy (Huynh et al., 2018; Diaz et al., 2020).

## 5 Conclusions

In conclusion, this study highlights the value of MAGIC populations in accelerating genetic gain through enhanced trait dissection and selection. Their broad genetic base, higher recombination rates, and increased phenotypic diversity facilitate the identification of superior lines that combine high protein content and grain yield. The identification of top-performing lines demonstrates the potential of MAGIC populations to simultaneously improve nutritional quality and yield stability, offering a powerful tool for breeding resilient cultivars, particularly under the environmental conditions evaluated in this study. Furthermore, the extensive genetic diversity and recombination within this population provide an excellent foundation for future genome-wide association studies (GWAS) and quantitative trait loci (QTL) mapping to dissect the genetic basis of yield, protein content, and other agronomic traits, thereby supporting targeted breeding strategies in faba bean.

As a next step, re-sequencing the top-performing lines using the published 13K faba bean SNP array could identify identity-by-descent (IBD) segments and generate marker-assisted selection (MAS) resources to accelerate breeding. In parallel, multi-location G×E trials in diverse environments will be conducted to evaluate the stability and adaptability of the selected lines.

## Data Availability

The raw data supporting the conclusions of this article will be made available by the authors, without undue reservation.
