# Supplementary material for: Harnessing faba bean MAGIC populations for enhanced protein content, yield, and agronomic performance in diverse environments
Source: Front Plant Sci. 2026 Feb 23;17:1731294. doi: 10.3389/fpls.2026.1731294 (PMC12968143; doi:10.3389/fpls.2026.1731294)
Supplement: Supplementary file 1 [file DataSheet1.docx]

Supplementary Material

# Supplementary Tables and Figures

## Supplementary Tables

**Supplementary Table 1.** Statistical model comparison of Automatic REML analysis (AIC/BIC) across traits and locations.

| **Statistical Model** | **Trait** | **Location** | **Random Model** | **Deviance** | **AIC** | **SIC** | **Random d.f.** |
| --- | --- | --- | --- | --- | --- | --- | --- |
| Row-by-column design | DFLR_G | Terbol | No spatial model | 5425.08 | 5433.08 | 5452.67 | 4 |
| Row-by-column design | DFLR_G | Terbol | I(x)power | 5422.23 | 5434.23 | 5463.62 | 6 |
| Row-by-column design | DFLR_S | Terbol | spatial model | 5339.5 | 5347.5 | 5367.09 | 4 |
| Row-by-column design | DFLR_S | Terbol | I(x)power | 5336.41 | 5348.41 | 5377.8 | 6 |
| Row-by-column design | DMAT_G | Terbol | spatial model | 5347.84 | 5355.84 | 5375.43 | 4 |
| Row-by-column design | DMAT_G | Terbol | power(x)I | 5344.43 | 5356.43 | 5385.81 | 6 |
| Row-by-column design | DMAT_G | Terbol | I(x)power | 5333.43 | 5345.43 | 5374.81 | 6 |
| Row-by-column design | DMAT_S | Terbol | spatial model | 5296.4 | 5304.4 | 5323.99 | 4 |
| Row-by-column design | DMAT_S | Terbol | I(x)power | 5282.87 | 5294.87 | 5324.25 | 6 |
| Row-by-column design | Protein_% | Terbol | No spatial model | 3377.27 | 3383.27 | 3397.96 | 3 |
| Incomplet×10-block design | BYPLT | Terbol | -REP_NO | 9030.29 | 9034.29 | 9043.99 | 2 |
| Incomplet×10-block design | BYPLT | Terbol | -REP_NO.BLK_NO | 9034.27 | 9038.27 | 9047.97 | 2 |
| Incomplet×10-block design | HSW | Terbol | -REP_NO | 6585.45 | 6589.45 | 6599.24 | 2 |
| Incomplet×10-block design | HSW | Terbol | -REP_NO.BLK_NO | 6586.27 | 6590.27 | 6600.06 | 2 |
| Incomplet×10-block design | NPP | Terbol | Blocks | 6215.18 | 6221.18 | 6235.87 | 3 |
| Incomplet×10-block design | GYPLT | Terbol | Blocks | 8011.86 | 8017.86 | 8032.54 | 3 |
| Incomplet×10-block design | NSPLT | Terbol | Blocks | 8553.42 | 8559.42 | 8574.1 | 3 |
| Incomplet×10-block design | NSP | Terbol | -REP_NO | 2767.23 | 2771.23 | 2781.01 | 2 |
| Incomplet×10-block design | NSP | Terbol | -REP_NO.BLK_NO | 2759.29 | 2763.29 | 2773.07 | 2 |
| Row-by-column design | DFLR_G | Marchouch | No spatial model | 5697.25 | 5705.25 | 5724.85 | 4 |
| Row-by-column design | DFLR_S | Marchouch | No spatial model | 5697.25 | 5705.25 | 5724.85 | 4 |
| Row-by-column design | DMAT_G | Marchouch | No spatial model | 5883.4 | 5891.4 | 5911 | 4 |
| Row-by-column design | DMAT_G | Marchouch | power(x)I | 5872.35 | 5884.35 | 5913.75 | 6 |
| Row-by-column design | DMAT_G | Marchouch | I(x)power | 5849.04 | 5861.04 | 5890.44 | 6 |
| Row-by-column design | DMAT_S | Marchouch | No spatial model | 5883.4 | 5891.4 | 5911 | 4 |
| Row-by-column design | DMAT_S | Marchouch | power(x)I | 5872.35 | 5884.35 | 5913.75 | 6 |
| Row-by-column design | DMAT_S | Marchouch | I(x)power | 5849.04 | 5861.04 | 5890.44 | 6 |
| Row-by-column design | HSW | Marchouch | No spatial model | 7291.83 | 7297.83 | 7312.45 | 3 |
| Row-by-column design | HSW | Marchouch | I(x)power | 7288.68 | 7298.68 | 7323.05 | 5 |
| Row-by-column design | NPP | Marchouch | No spatial model | 4954.95 | 4960.95 | 4975.6 | 3 |
| Row-by-column design | NPP | Marchouch | power(x)I | 4942.1 | 4952.1 | 4976.52 | 5 |
| Row-by-column design | NSP | Marchouch | Replicates & Rows & Columns | 1191.34 | 1199.34 | 1218.88 | 4 |
| Row-by-column design | NSP | Marchouch | Replicates & Rows & Columns & I(x)power | 1188.16 | 1200.16 | 1229.48 | 6 |
| Row-by-column design | NSP | Marchouch | Replicates & Rows & Columns & I(x)power & measurement_error | 1188.16 | 1200.16 | 1229.48 | 6 |
| Row-by-column design | GYPLT | Marchouch | No spatial model | 5486.1 | 5492.1 | 5506.47 | 3 |
| Row-by-column design | GYPLT | Marchouch | power(x)I | 5469.14 | 5479.14 | 5503.09 | 5 |
| Row-by-column design | Protein_% | Marchouch | No spatial model | 1976.33 | 1982.33 | 1994.63 | 3 |
| Incomplet×10-block design | BYPLT | Marchouch | Blocks | 6461.95 | 6467.95 | 6482.26 | 3 |
| Incomplet×10-block design | NSPLT | Marchouch | -ROW_NO-ROW_NO.BLK_NO | 6923.51 | 6929.51 | 6944.17 | 3 |
| Incomplet×10-block design | NSPLT | Marchouch | -ROW_NO-REP_NO | 6870.19 | 6876.19 | 6890.86 | 3 |
| Incomplet×10-block design | NSPLT | Marchouch | -ROW_NO-BLK_NO.REP_NO | 6871.4 | 6877.4 | 6892.06 | 3 |
| Incomplet×10-block design | NSPLT | Marchouch | -ROW_NO.BLK_NO-REP_NO | 6855.29 | 6861.29 | 6875.96 | 3 |
| Incomplet×10-block design | NSPLT | Marchouch | -ROW_NO.BLK_NO-BLK_NO.REP_NO | 6855.95 | 6861.95 | 6876.62 | 3 |

**Supplementary Table 2.** Correlation analysis of different traits evaluated in MAGIC population at Marchouch and Terbol during 2023-2024 growing season.

| **Marchouch** | | | | | **Terbol** | | | | |
| --- | --- | --- | --- | --- | --- | --- | --- | --- | --- |
| **Trait1** | **Trait2** | **r** | **p_value** | **q_value** | **Trait1** | **Trait2** | **r** | **p_value** | **q_value** |
| DMAT_G | DFLR_G | 0.405399 | 4.23×10^-97^ | 2.12×10^-96^ | DFLR_G | DMAT_G | 0.34143 | 0.0000 | 0.0000 |
| DMAT_G | PLHT | 0.457789 | 1.39×10^-126^ | 7.84×10^-126^ | DFLR_G | PLHT | 0.034396 | 0.089710701 | 0.106236357 |
| DFLR_G | PLHT | 0.189839 | 3.18×10^-21^ | 5.72×10^-21^ | DMAT_G | PLHT | 0.116358 | 8.49×10^-09^ | 1.53×10^-08^ |
| DMAT_G | BYPLT | 0.122293 | 1.83×10^-09^ | 2.66×10^-09^ | DFLR_G | BYPLT | 0.022742 | 0.264705578 | 0.290530512 |
| DFLR_G | BYPLT | -0.02308 | 0.258084372 | 0.265681274 | DMAT_G | BYPLT | 0.160243 | 2.66×10^-15^ | 6.31×10^-15^ |
| PLHT | BYPLT | 0.253829 | 1.25×10^-36^ | 2.96×10^-36^ | PLHT | BYPLT | 0.214255 | 0.0000 | 0.0000 |
| DMAT_G | GYPLT | 0.003947 | 0.846617182 | 0.846617182 | DFLR_G | GYPLT | -0.11 | 5.31×10^-08^ | 8.53×10^-08^ |
| DFLR_G | GYPLT | -0.2431 | 1.09×10^-33^ | 2.45×10^-33^ | DMAT_G | GYPLT | 0.115689 | 1.04×10^-08^ | 1.80×10^-08^ |
| PLHT | GYPLT | 0.145285 | 8.12×10^-13^ | 1.22×10^-12^ | PLHT | GYPLT | 0.238181 | 0.0000 | 0.0000 |
| BYPLT | GYPLT | 0.626 | 1.33×10^-258^ | 1.50×10^-257^ | BYPLT | GYPLT | 0.673019 | 0.0000 | 0.0000 |
| DMAT_G | NPPLT | -0.23064 | 1.47×10^-30^ | 3.01×10^-30^ | DFLR_G | NPPLT | -0.00487 | 0.810174633 | 0.828587693 |
| DFLR_G | NPPLT | -0.23596 | 5.85×10^-32^ | 1.25×10^-31^ | DMAT_G | NPPLT | -0.01864 | 0.358041846 | 0.383616264 |
| PLHT | NPPLT | -0.04225 | 0.037719258 | 0.042434165 | PLHT | NPPLT | 0.063674 | 0.001672324 | 0.002351706 |
| BYPLT | NPPLT | 0.484051 | 1.84×10^-140^ | 1.18×10^-139^ | BYPLT | NPPLT | 0.148268 | 2.66×10^-13^ | 5.69×10^-13^ |
| GYPLT | NPPLT | 0.654503 | 8.50×10^-294^ | 1.28×10^-292^ | GYPLT | NPPLT | 0.240217 | 0.0000 | 0.0000 |
| DMAT_G | NSPLT | -0.31328 | 1.96×10^-56^ | 6.79×10^-56^ | DFLR_G | NSPLT | -0.12502 | 6.46×10^-10^ | 1.21×10^-09^ |
| DFLR_G | NSPLT | -0.27172 | 2.25×10^-42^ | 6.32×10^-42^ | DMAT_G | NSPLT | -0.14718 | 3.21×10^-13^ | 6.56×10^-13^ |
| PLHT | NSPLT | -0.07505 | 0.000213708 | 0.000253075 | PLHT | NSPLT | 0.094339 | 3.25×10^-06^ | 5.04×10^-06^ |
| BYPLT | NSPLT | 0.538973 | 1.19×10^-180^ | 1.07×10^-179^ | BYPLT | NSPLT | 0.451347 | 0.0000 | 0.0000 |
| GYPLT | NSPLT | 0.663487 | 4.33×10^-305^ | 9.75×10^-304^ | GYPLT | NSPLT | 0.742105 | 0.0000 | 0.0000 |
| NPPLT | NSPLT | 0.850443 | 0.0000 | 0.0000 | NPPLT | NSPLT | 0.29352 | 0.0000 | 0.0000 |
| DMAT_G | NSP | -0.16762 | 1.01×10^-16^ | 1.62×10^-16^ | DFLR_G | NSP | -0.11248 | 2.95×10^-08^ | 4.91×10^-08^ |
| DFLR_G | NSP | -0.17883 | 7.34×10^-19^ | 1.27×10^-18^ | DMAT_G | NSP | -0.1254 | 6.17×10^-10^ | 1.21×10^-09^ |
| PLHT | NSP | -0.04065 | 0.045424907 | 0.049856605 | PLHT | NSP | 0.053464 | 0.008564561 | 0.011335448 |
| BYPLT | NSP | 0.212089 | 9.92×10^-26^ | 1.94×10^-25^ | BYPLT | NSP | 0.32912 | 0.0000 | 0.0000 |
| GYPLT | NSP | 0.27625 | 2.22×10^-43^ | 7.12×10^-43^ | GYPLT | NSP | 0.5428 | 0.0000 | 0.0000 |
| NPPLT | NSP | 0.196908 | 1.46×10^-22^ | 2.74×10^-22^ | NPPLT | NSP | -0.3442 | 0.0000 | 0.0000 |
| NSPLT | NSP | 0.340257 | 9.80×10^-67^ | 4.01×10^-66^ | NSPLT | NSP | 0.748382 | 0.0000 | 0.0000 |
| DMAT_G | HSW | 0.501096 | 3.17×10^-154^ | 2.38×10^-153^ | DFLR_G | HSW | 0.070346 | 0.00052277 | 0.000758859 |
| DFLR_G | HSW | 0.105146 | 2.12×10^-07^ | 2.98×10^-07^ | DMAT_G | HSW | 0.371648 | 0.0000 | 0.0000 |
| PLHT | HSW | 0.383142 | 1.35×10^-85^ | 6.06×10^-85^ | PLHT | HSW | 0.209769 | 0.0000 | 0.0000 |
| BYPLT | HSW | 0.270425 | 2.41×10^-41^ | 6.03×10^-41^ | BYPLT | HSW | 0.27293 | 0.0000 | 0.0000 |
| GYPLT | HSW | 0.275332 | 5.24×10^-43^ | 1.57×10^-42^ | GYPLT | HSW | 0.282235 | 0.0000 | 0.0000 |
| NPPLT | HSW | -0.26968 | 1.69×10^-41^ | 4.49×10-41 | NPPLT | HSW | -0.07185 | 0.000394541 | 0.000591812 |
| NSPLT | HSW | -0.33713 | 1.69×10^-65^ | 6.34×10^-65^ | NSPLT | HSW | -0.3808 | 0.0000 | 0.0000 |
| NSP | HSW | -0.08314 | 4.25×10^-05^ | 5.17×10^-05^ | NSP | HSW | -0.31461 | 0.0000 | 0.0000 |
| DMAT_G | Protein | -0.15196 | 7.22×10^-14^ | 1.12×10^-13^ | DFLR_G | Protein | -0.15159 | 5.48×10^-14^ | 1.23×10^-13^ |
| DFLR_G | Protein | -0.1773 | 2.09×10^-18^ | 3.48×10^-18^ | DMAT_G | Protein | 0.004995 | 0.805414124 | 0.828587693 |
| PLHT | Protein | -0.09844 | 1.35×10^-06^ | 1.78×10^-06^ | PLHT | Protein | 0.02957 | 0.144643405 | 0.162723831 |
| BYPLT | Protein | -0.02316 | 0.259777245 | 0.265681274 | BYPLT | Protein | -0.00141 | 0.94478166 | 0.94478166 |
| GYPLT | Protein | 0.028804 | 0.160010452 | 0.17143977 | GYPLT | Protein | 0.046971 | 0.020429124 | 0.025536405 |
| NPPLT | Protein | 0.06467 | 0.0015462 | 0.001784076 | NPPLT | Protein | -0.03101 | 0.126104056 | 0.14550468 |
| NSPLT | Protein | 0.088186 | 1.51×10^-05^ | 1.89×10^-05^ | NSPLT | Protein | 0.048078 | 0.017828156 | 0.022921915 |
| NSP | Protein | 0.088524 | 1.41×10^-05^ | 1.81×10^-05^ | NSP | Protein | 0.062093 | 0.002248289 | 0.003065849 |
| HSW | Protein | -0.10097 | 7.34×10^-07^ | 1.00×10^-06^ | HSW | Protein | -0.03444 | 0.089593056 | 0.106236357 |

r, Person coefficient of correlation; p_value, original p_values; q-value, Benjamini–Hochberg FDR-adjusted significance (αFDR = 0.05).

## Supplementary Figures

**Supplementary Figure 1.** Residual diagnostic plots for all traits evaluated at Terbol during the 2023–2024 growing season.


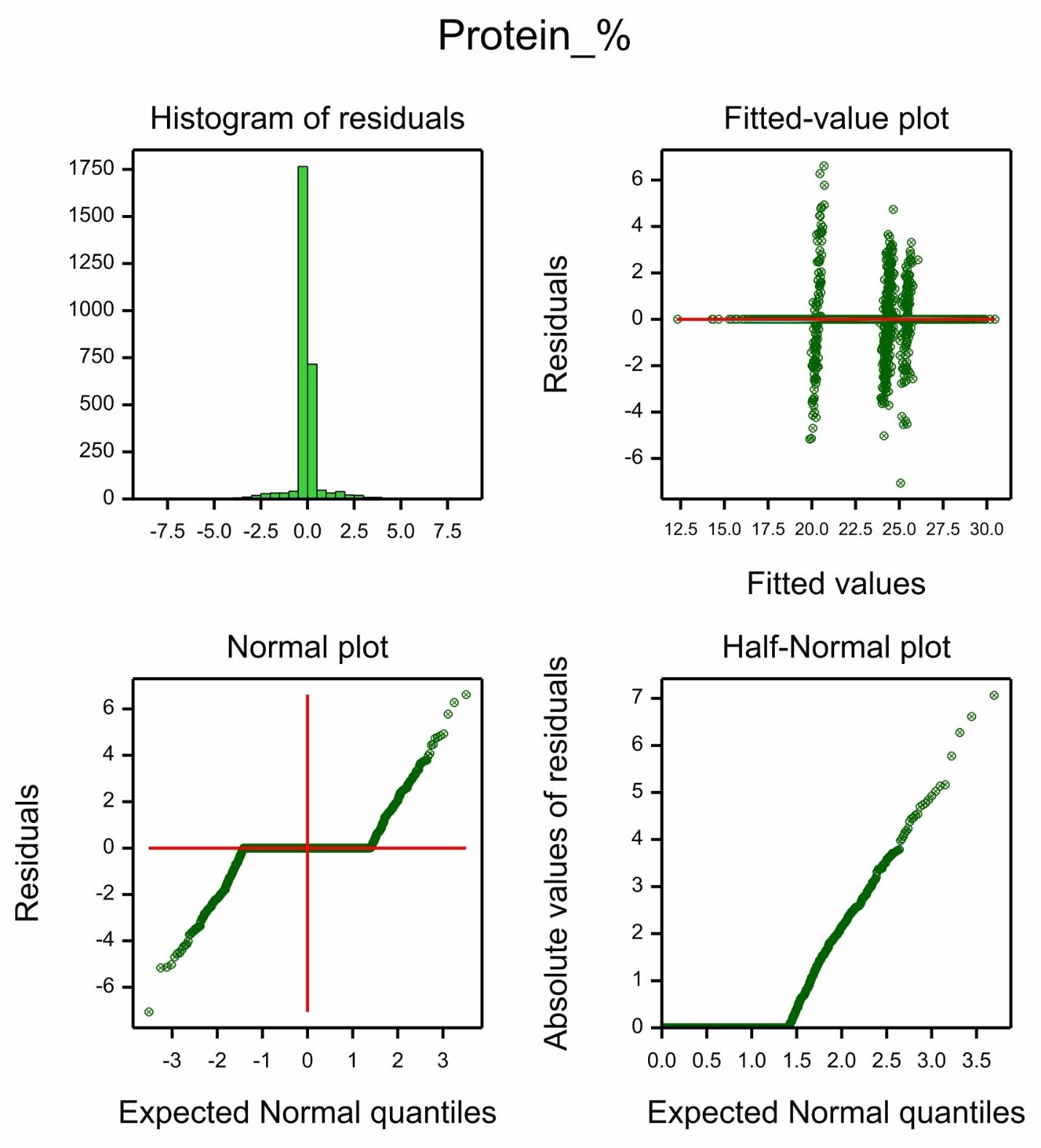


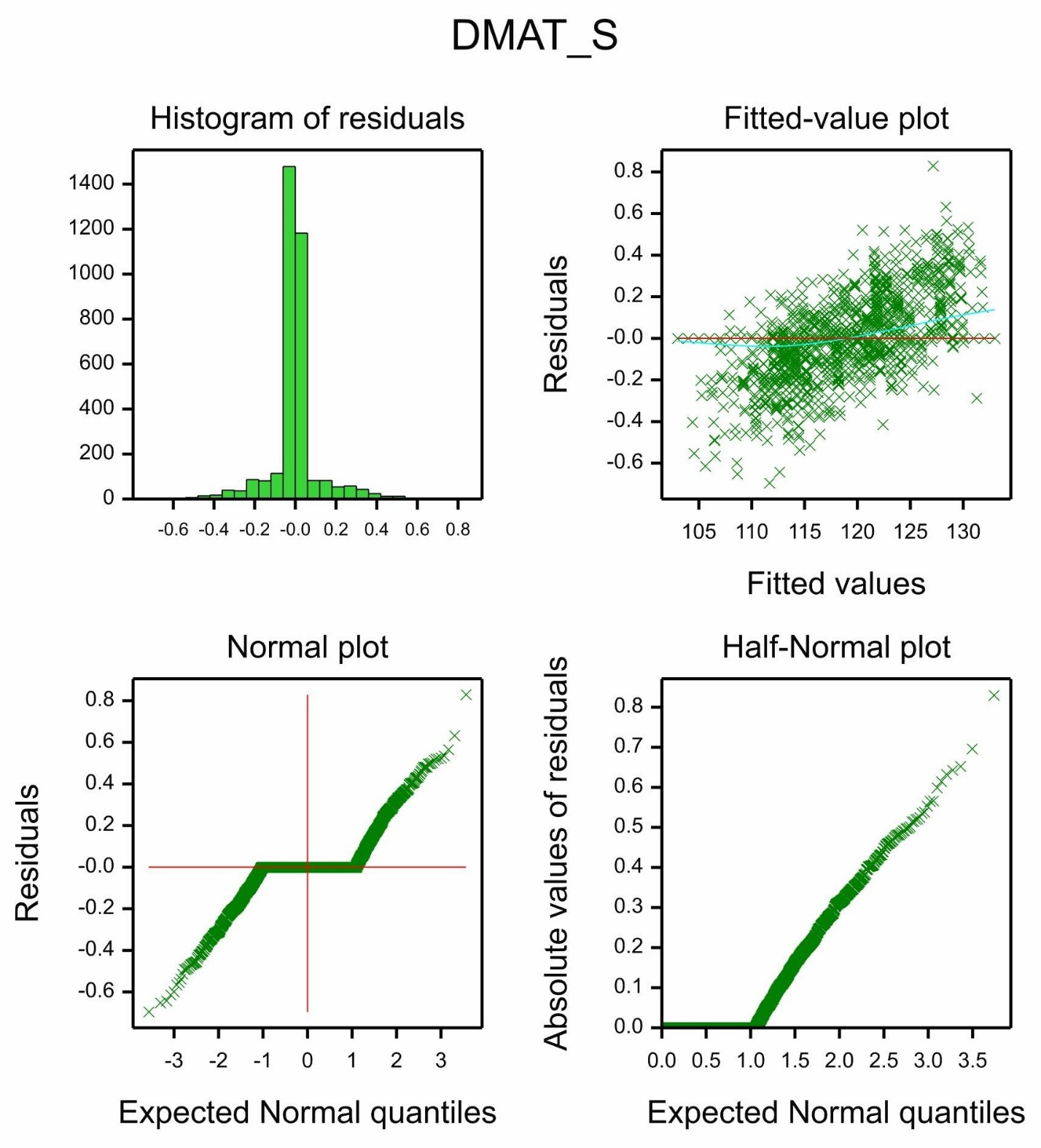


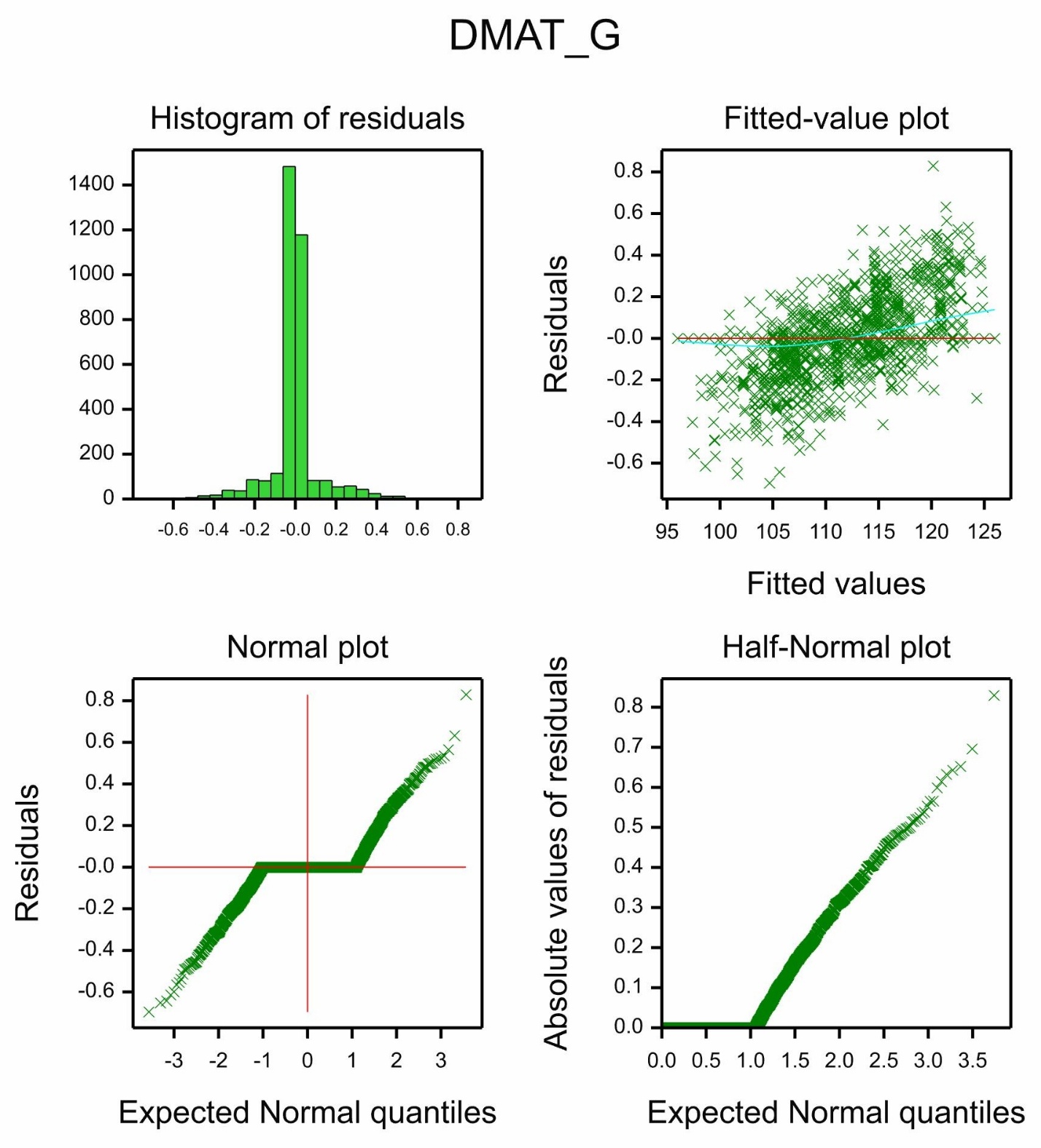


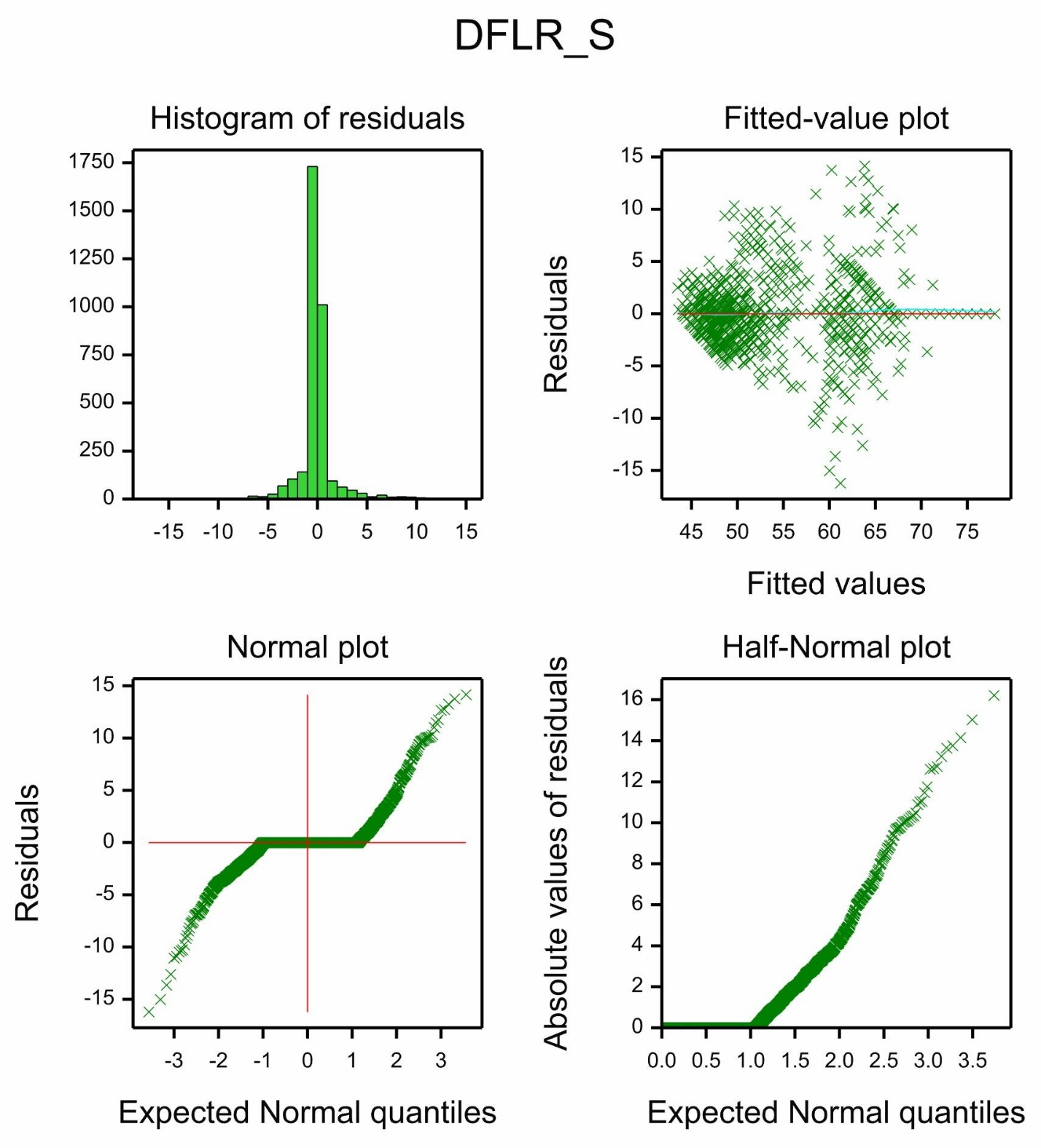


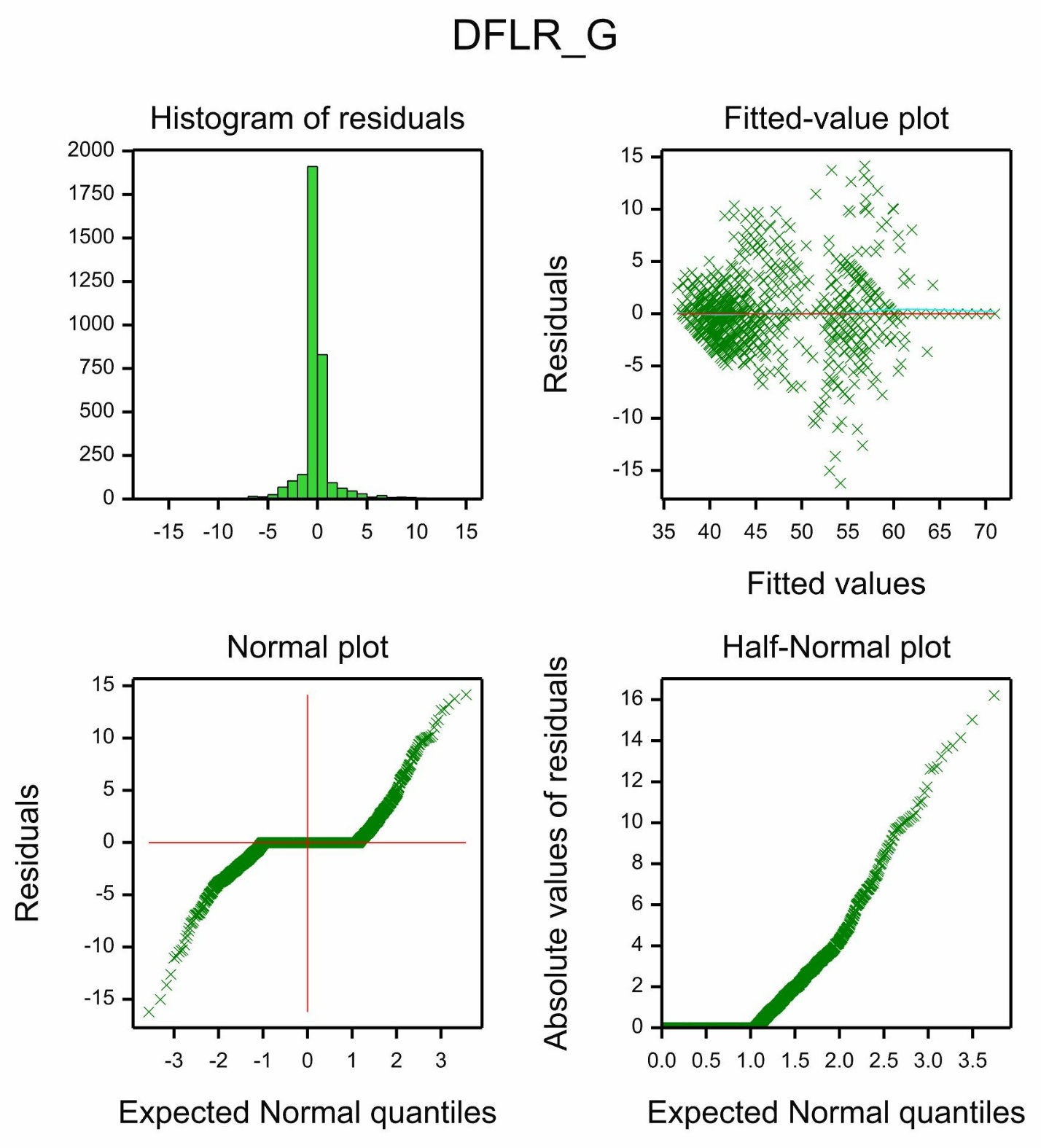


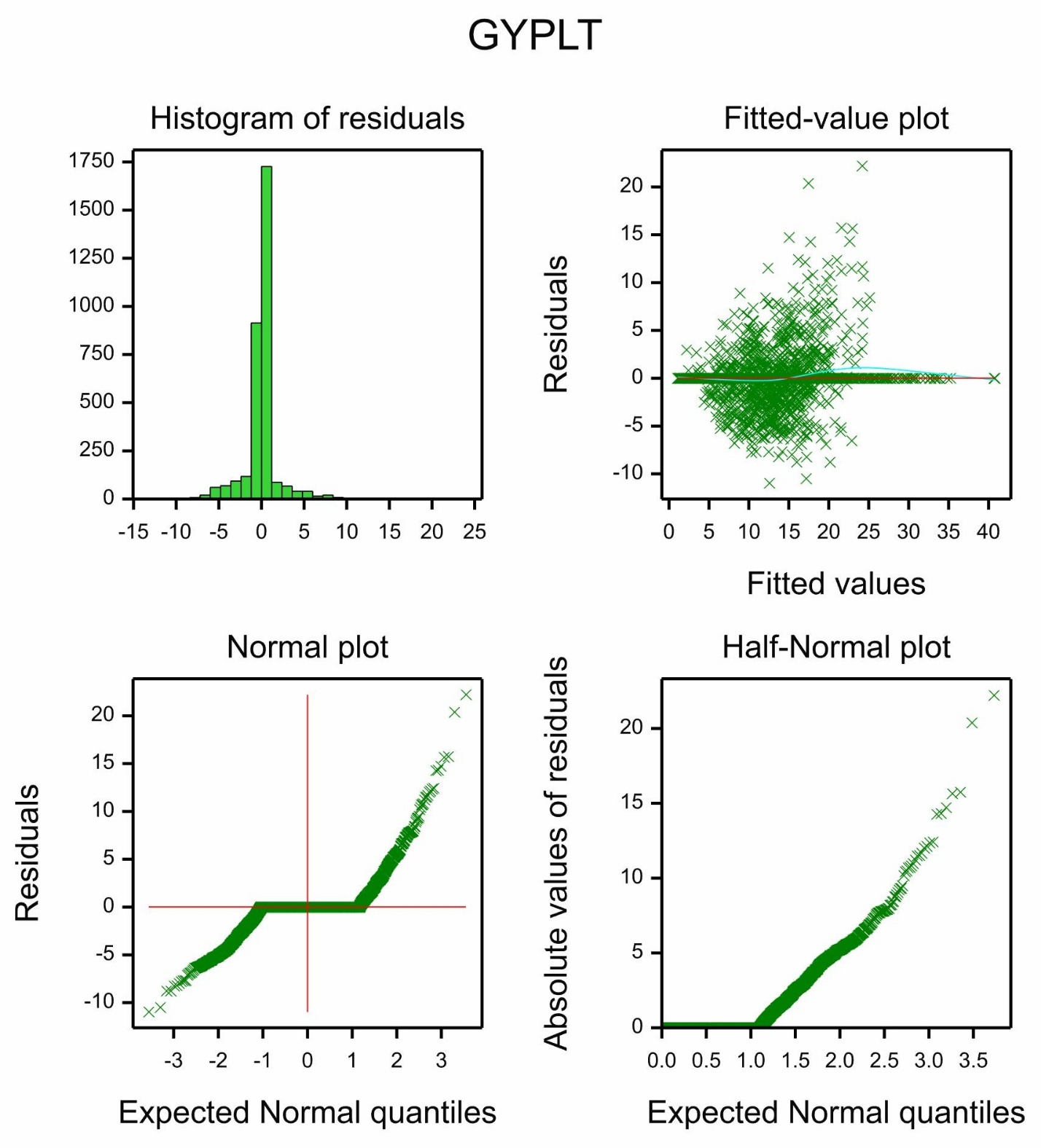


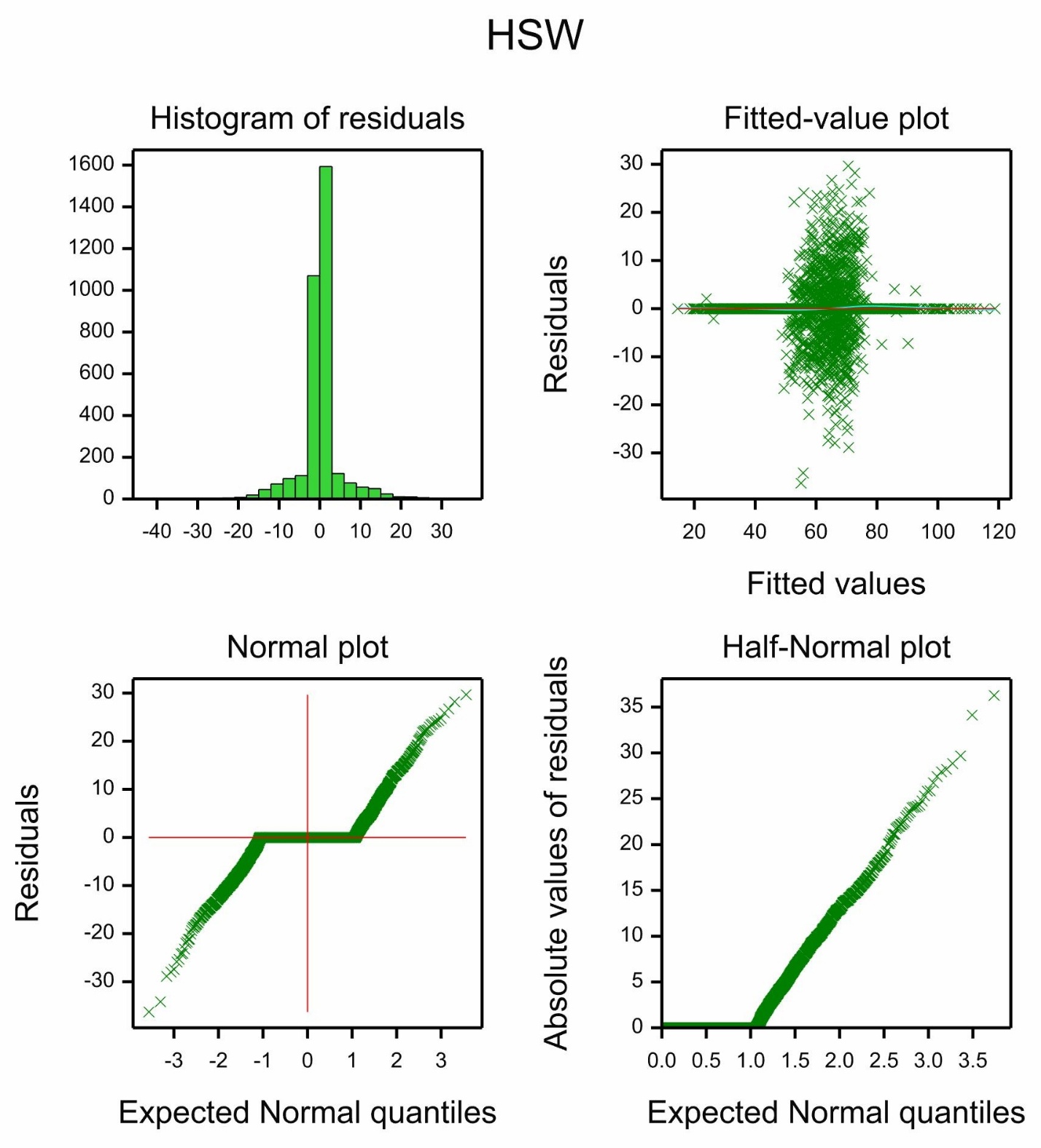


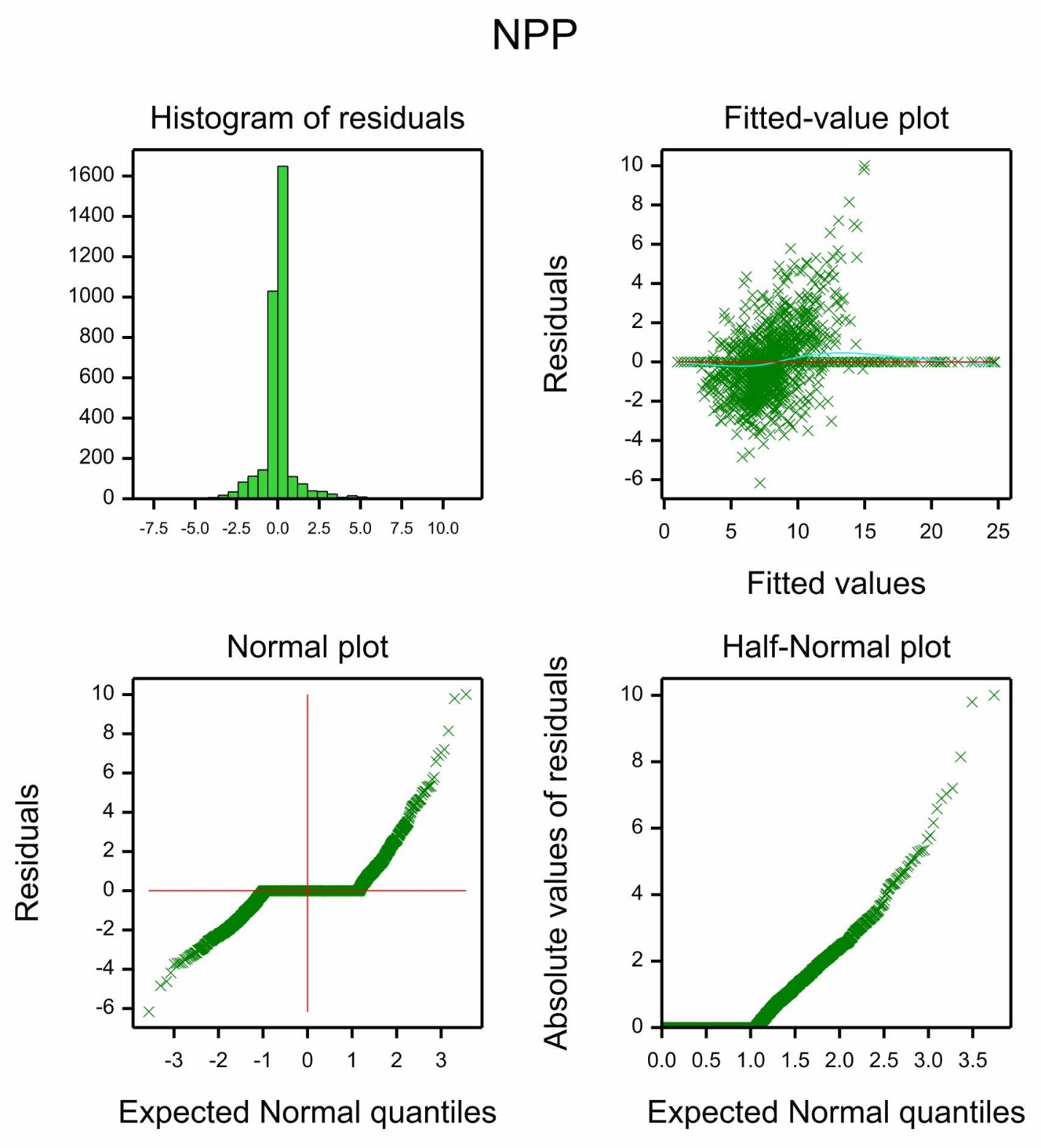


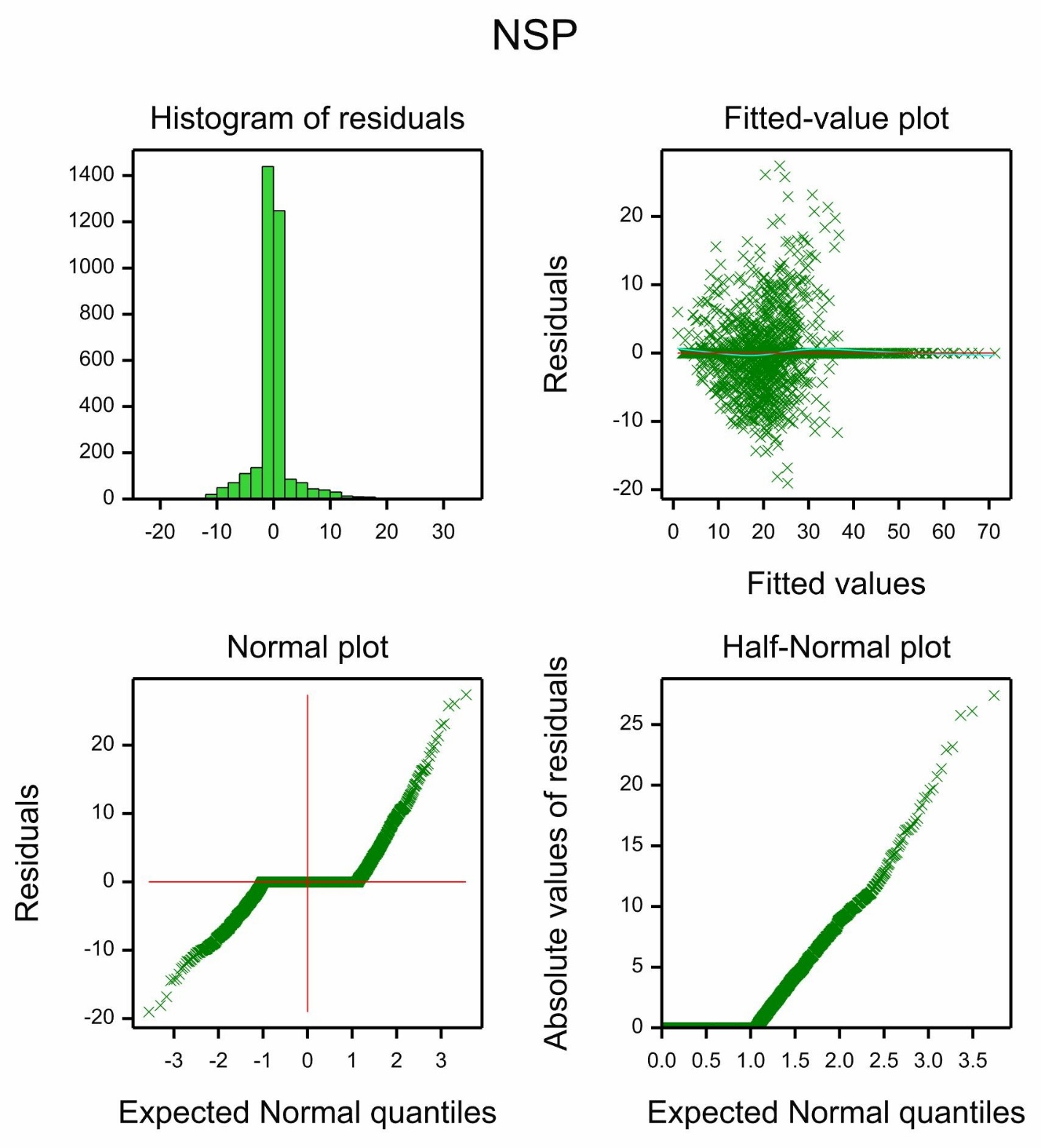


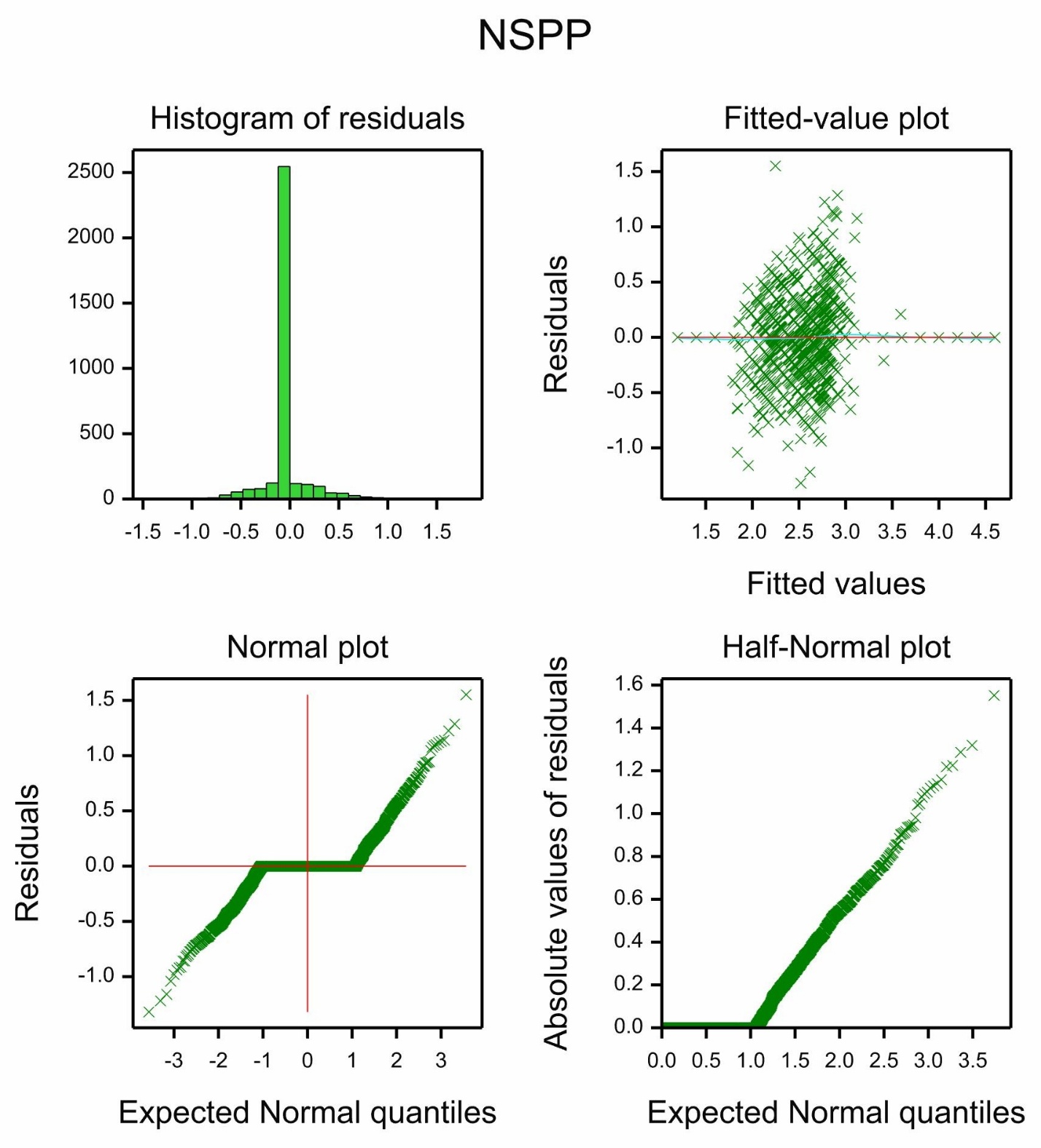


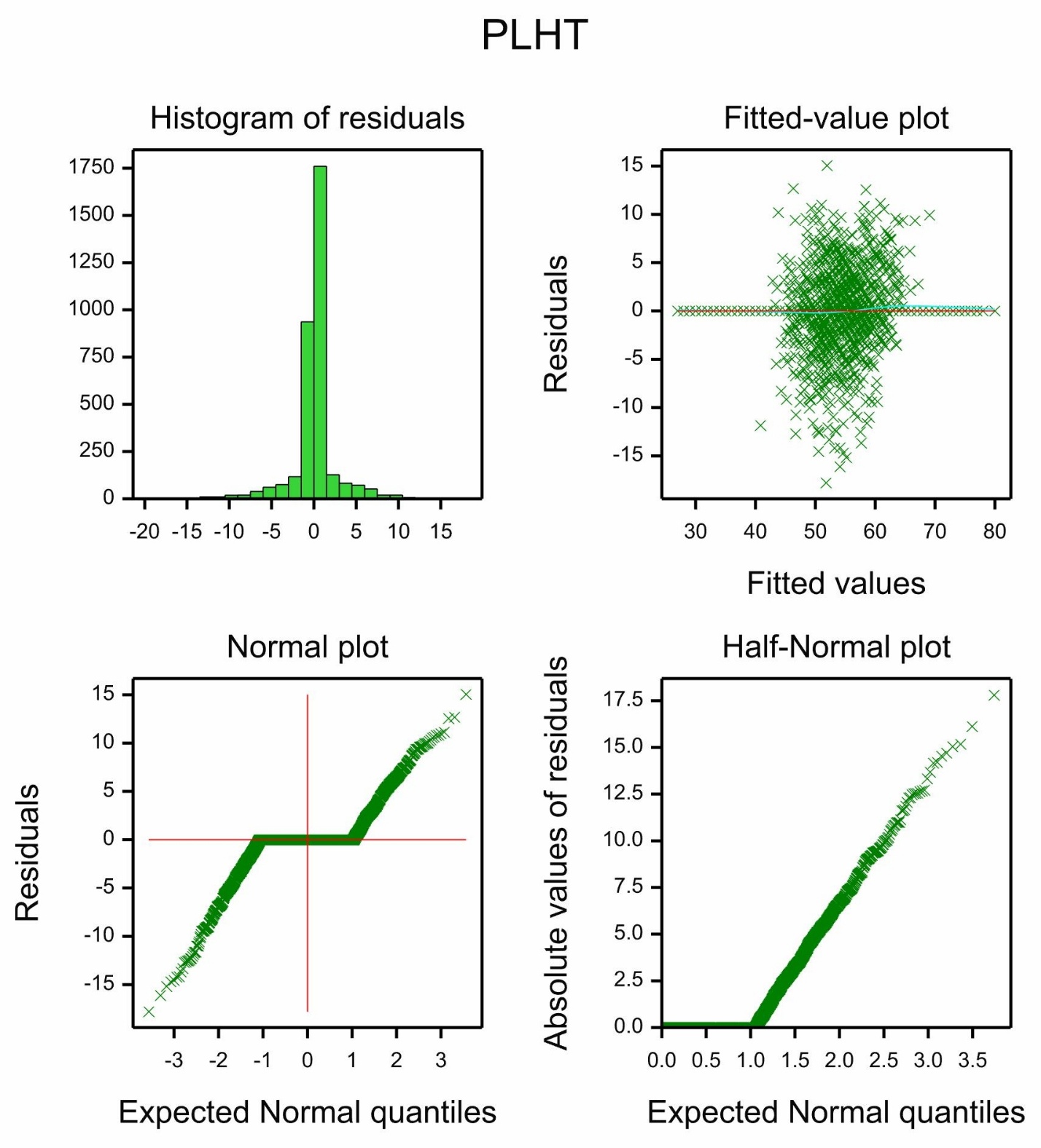


**Supplementary Figure 2.** Residual diagnostic plots for all traits evaluated at Marchouch during the 2023–2024 growing season.


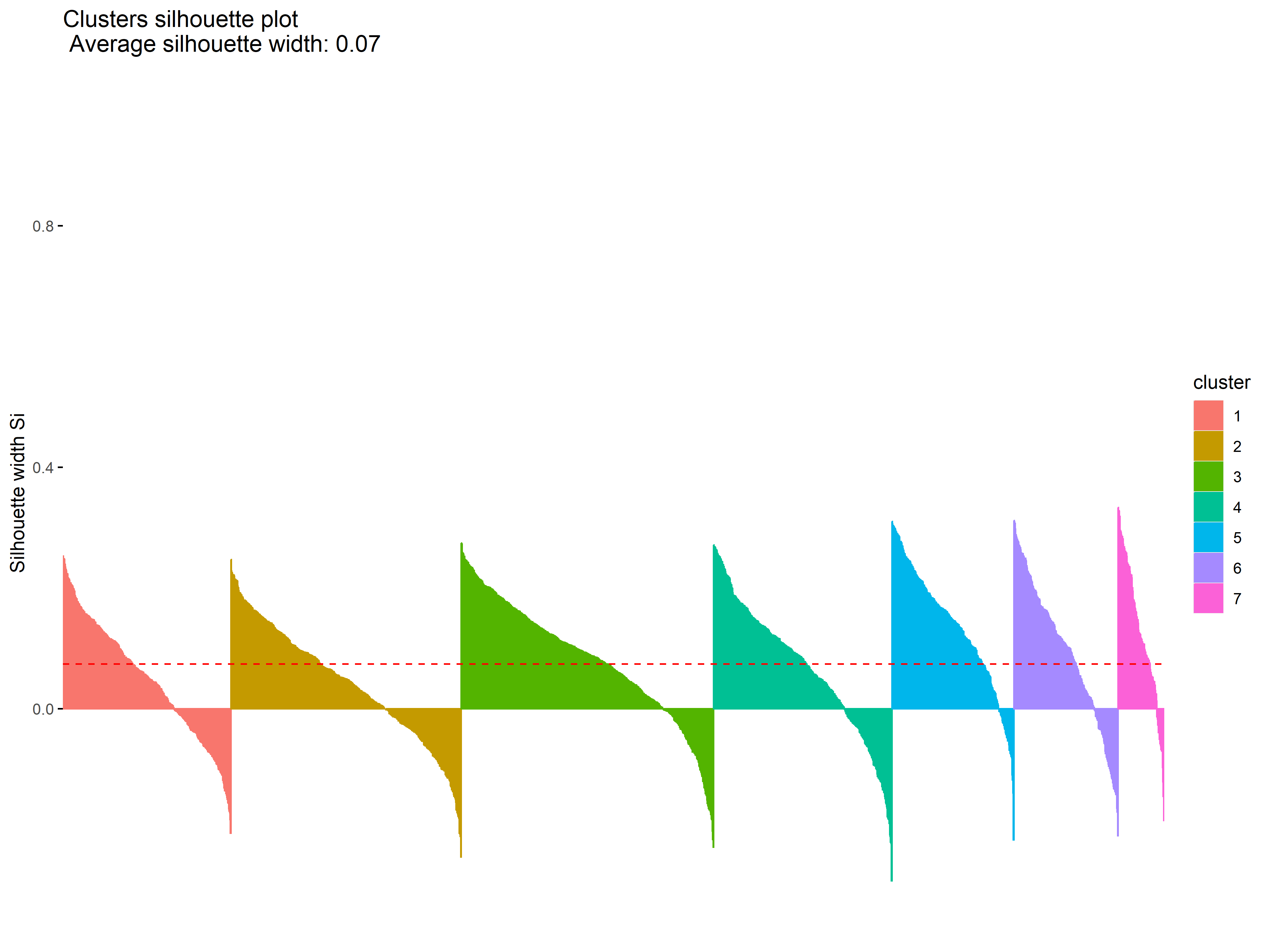


**Supplementary Figure 3.** Silhouette plot for Terbol location during 2023-2024 growing season.


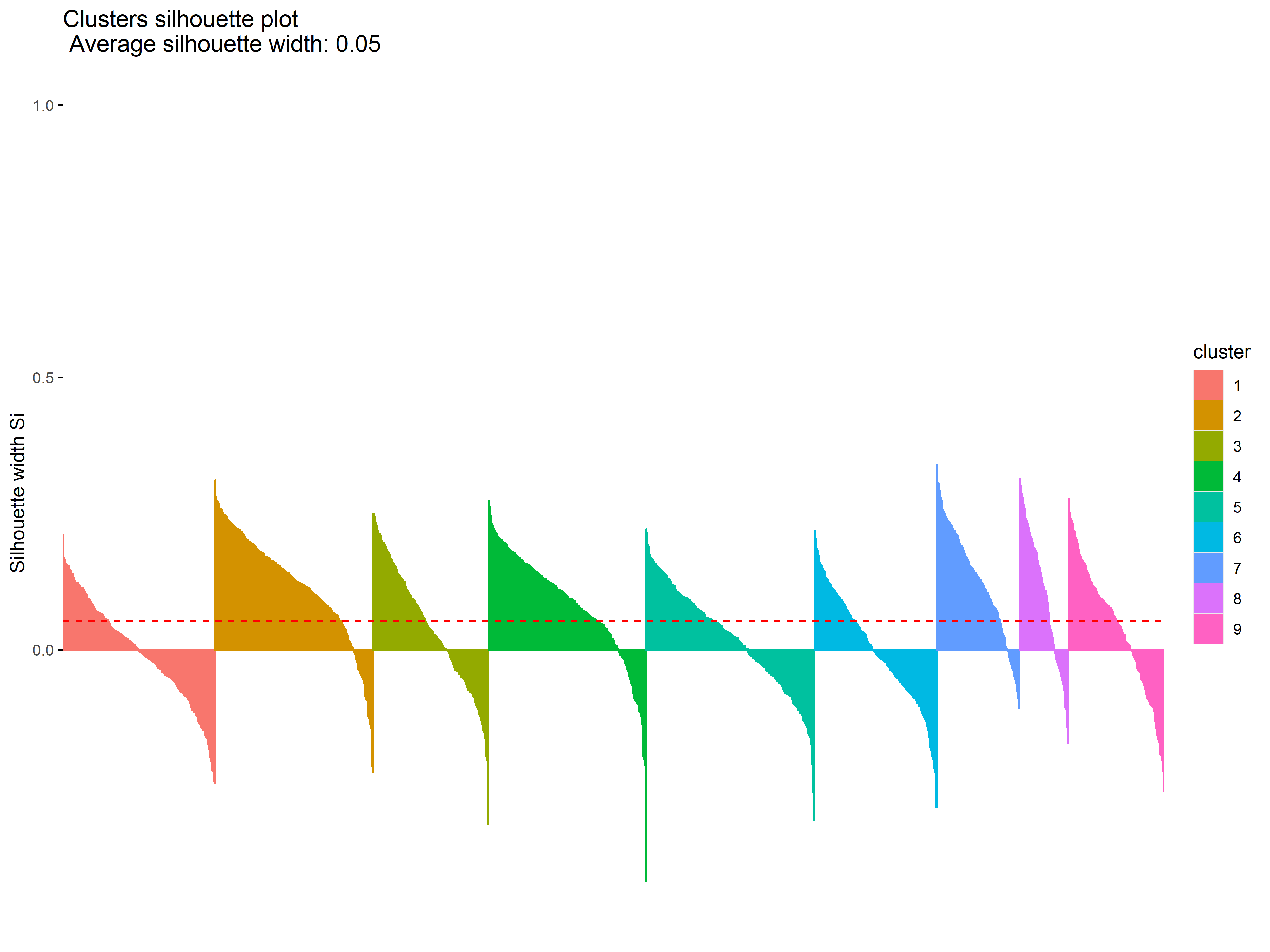


**Supplementary Figure 4.** Silhouette plot for Marchouch location during 2023-2024 growing season.


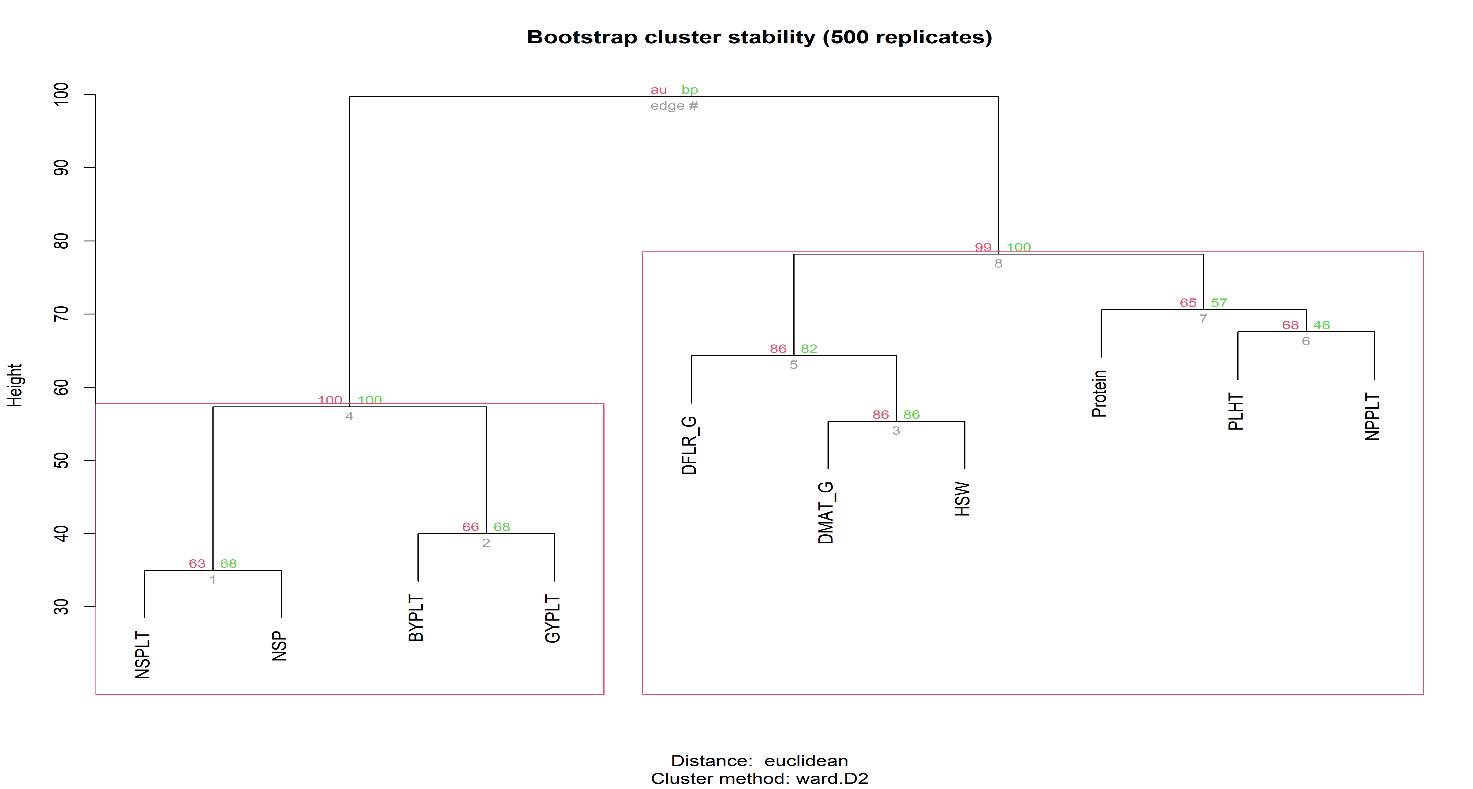


**Supplementary Figure 5.** Bootstrap cluster stability for Terbol during 2023-2024 growing season.

**
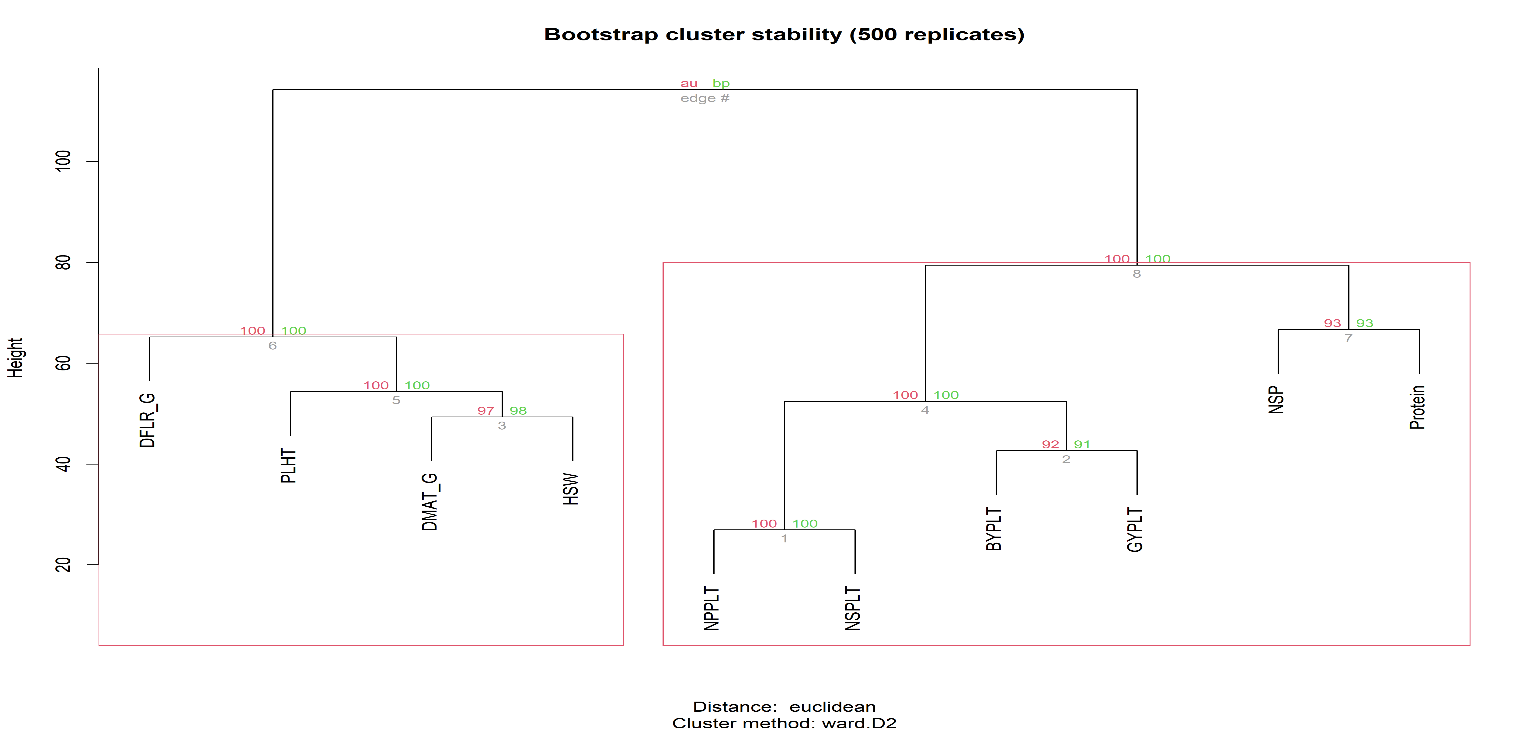
**

**Supplementary Figure 6.** Bootstrap cluster stability for Marchouch during 2023-2024 growing season.
